# Rapid Activation of 3D-Printed Carbon Electrodes by Atmospheric Air Plasma: Toward Electrochemical Drug Analysis

**DOI:** 10.1021/acsomega.5c05879

**Published:** 2025-08-23

**Authors:** Miroslav Kováč, Katarína Gregová, Ĺubomíŕ́ Švorc, František Zažímal, Tomáš Homola, Pavol Gemeiner

**Affiliations:** † Department of Graphic Arts Technology and Applied Photochemistry, Faculty of Chemical and Food Technology, 201272 Slovak University of Technology in Bratislava, Radlinského 9, 812 37 Bratislava, Slovakia; ‡ Institute of Analytical Chemistry, Faculty of Chemical and Food Technology, 201272 Slovak University of Technology in Bratislava, Radlinského 9, 812 37 Bratislava, Slovakia; § Department of Plasma Physics and Technology, Faculty of Science, 37748 Masaryk University, Kotlářská 267/2, 602 00 Brno, Czech Republic

## Abstract

This study presents a rapid, environmentally friendly, and scalable activation method for 3D-printed poly­(lactic acid)/carbon black (PLA/CB) electrodes using atmospheric air plasma under ambient conditions. The goal was to optimize the plasma activation time and compare its efficiency with conventional activation techniques using *N*,*N*-dimethylformamide (DMF) and sodium hydroxide (NaOH). Surface morphology, chemical composition, wettability, and electrochemical performance were systematically evaluated through scanning electron microscopy (SEM), Raman spectroscopy, XPS, contact angle measurements, cyclic voltammetry (CV), and electrochemical impedance spectroscopy (EIS). Plasma treatment, as short as 5 s, effectively removed the PLA matrix from the electrode surface, enhanced surface roughness, hydrophilicity, and exposure of conductive carbon black particles, leading to increased electrochemical performance. Compared to chemical activation, 40 s of plasma activation yielded comparable performance with significantly shorter processing times (vs NaOH) and without hazardous solvents (such as DMF). Finally, the activated electrodes were successfully applied in the development, optimization, and validation of a novel electrochemical protocol for the determination of the antihypertensive drug amlodipine, revealing high sensitivity, a low limit of detection of 0.09 μM, precision (RSD of 6.6%), and recovery (97.1 and 105.4%) in pharmaceutical formulations. The findings demonstrate the promising potential of air plasma activation as a sustainable and efficient approach for preparing 3D-printed electrodes for analytical and sensing applications.

## Graphical abstract

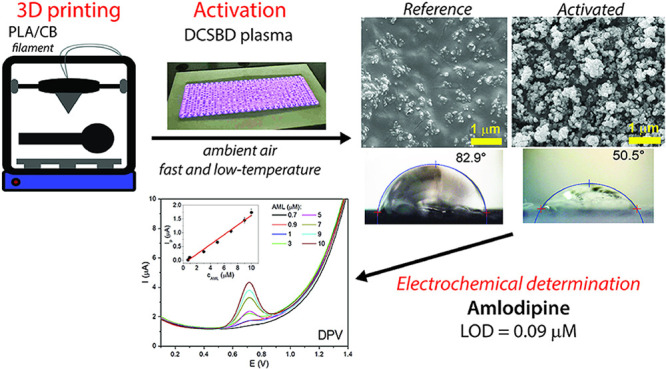

## Introduction

Additive manufacturing (3D printing) has grown in popularity since the mid-1980s, with stereolithography and fused deposition modeling (FDM) being prominent methods. FDM technology, for example, uses polymer filaments such as poly­(lactic acid) (PLA), polyethylene (PE), and acrylonitrile butadiene styrene (ABS) to create models layer by layer. While metal 3D printing is expensive, polymer-based printing, especially using carbon-loaded filaments, represents a cost-effective solution for producing electrochemical sensors.^1–3^ Despite its advantages, FDM presents limitations such as high porosity and poor sealing, which compromise the quality of printed components.^4,5^ To address these issues, reported studies have focused on optimizing printing parameters, including infill, layer height, printing orientation, and temperature.^5–7^ These significantly influence the mechanical, morphological, and structural properties of 3D-printed electrochemical sensors.

In 3D printing, the filament is melted and applied layer by layer, with the print head moving over multiple axes. The growing interest in biopolymers, such as PLA, stems from ecological concerns and its complete biodegradability under controlled industrial conditions. PLA, made from renewable resources, such as carbohydrates, is an environmentally friendly alternative to petroleum-based polymers. It is used in various applications, including biomedical fields.^8–11^

Recently, the applications of 3D printing in the development of miniaturized electrochemical sensors and biosensors have been intensively studied.^12–14^ Electrochemical sensors operate by detecting reactions at the electrode/solution interface. Traditional working electrode materials, such as those made from glassy carbon, gold, or platinum,^15^ are often costly, prone to surface contamination, and have limited reproducibility.^16^ In contrast, 3D-printed electrodes offer low-cost production, design flexibility, ease of surface modification, and high potential for scalable and disposable sensors.^17,18^

3D-printed electrodes are produced using conductive filaments composed of a thermoplastic matrix and conductive filler. Although metallic fillers, such as copper^19^ and silver,^20^ have relatively lower electrical resistivity, carbon-based fillers provide several advantages, including: (*i*) carbon-based polymer composites typically do not need additional processing steps after printing, such as annealing, (*ii*) they can be easily incorporated into a filament form using low-cost filament extrusion device, and (*iii*) they have significantly longer shelf life and stability.^21,22^

Conductive carbon-based particles and nanofillers such as carbon black,^23^ graphene,^24^ graphene oxide,^25^ biochar,^26^ and carbon nanotubes^27^ are commonly incorporated into 3D printing PLA filaments, which were subsequently used as working electrodes in electrochemical sensing. Widely used commercial filaments include Black Magic (graphene-based) and Proto-Pasta (carbon black-based), both of which contain about 90% PLA.^28,18^ However, the resulting electrodes require surface activation to remove excess polymer, otherwise hindering the electrochemical performance. Several activation techniques were successfully studied, including chemical,^29,30^ electrochemical,^31,32^ mechanical,^33^ and thermal methods,^34^ though many of them are time-consuming, costly, or environmentally harmful. On the other hand, atmospheric cold plasma activation represents a faster, greener alternative with comparable effects on electrochemical properties.^29^ Laser-induced activation proceeds at a pace similar to plasma treatment, completing the surface modification in only seconds to a few minutes, considerably faster than the ∼10 min chemical activation.^35^

As a standard, chemical activation methods typically employ solvents like DMF or acetone, which swell the PLA matrix and promote its detachment from the electrode surface.^36^ NaOH treatment induces saponification (alkaline hydrolysis).^37^ Gusmão et al. demonstrated that DMF caused the most pronounced etching, followed by acetone, while methanol, ethanol, and water were ineffective. Acetone-treated electrodes exhibited faster electron transfer due to greater surface roughness.^36^ Redondo et al. applied NaBH_4_, which reduced PLA, achieving electrochemical properties comparable to DMF-activated electrodes.^38^ Thermal activation, explored by Novotný et al., involved heating PLA/graphene electrodes at 350 °C in an inert atmosphere for 3 h, leading to polymer removal without damaging the structure. The treatment enhanced electrochemical activity beyond that of DMF-activated electrodes.^39^ Dos Santos et al. investigated electrochemical activation by applying ±1.8 V for 900 s in phosphate buffer (pH 7.4), improving the overall electrode performance. Activated electrodes successfully detected dopamine.^40^ Kalinke et al. combined chemical (1 M NaOH, 30 min) and electrochemical (1.8 V, 900 s) treatments, achieving superior exposure of conductive particles and improved response signal for caffeic acid. Raman spectroscopy indicated a reduction of oxygen groups, further enhancing electrochemical properties.^30^ Finally, plasma treatment (cold reactive O_2_ and CO_2_ plasma treatment with a custom-designed microwave plasma-enhanced chemical vapor deposition system), as shown by Pereira et al., offers rapid activation without chemicals. PLA/CB electrodes exposed to plasma in a pure O_2_ and CO_2_ atmosphere for 2 min exhibited enhanced electrochemical behavior, confirmed by dopamine detection, demonstrating improved sensitivity and detection limits.^46^ The disadvantage of their plasma method was that a vacuum chamber with an argon gas supply was needed due to the excitation of the discharge at room temperature. Fontana-Escartín et al. used low-pressure radiofrequency plasma in the atmosphere of N_2_, O_2_, and air. Similarly, their method requested a closed chamber with controlled 0.8 mbar low pressure, and all activations lasted 2 min.^41^

In this work, atmospheric air plasma activation of 3D-printed PLA/carbon black working electrodes in the open chamber under ambient laboratory conditions was undertaken. The advantages of the plasma consist of its low temperature (<70 °C), high power density, the possibility of preparing plasma electrodes in large areas, and its application in roll-to-roll production.^42^ The plasma activation with standard chemical wet-activations in DMF and NaOH was compared. In addition, we aimed to evaluate and optimize plasma activation duration, regarding its impact on surface morphology and functionalization, wettability, and electrochemical performance compared with more traditional chemical activation methods. By systematically varying treatment duration and characterizing the resulting surfaces using SEM, Raman spectroscopy, XPS, contact angle, and electrochemical (CV, EIS) measurements, we seek to identify an efficient, scalable, and environmentally sustainable activation procedure. The overarching goal was to enhance the electrochemical activity of low-cost, 3D-printed electrodes for future application in miniaturized sensing platforms by a nontoxic, rapid, low-cost, mass-production-applicable activation method. Finally, developing, optimizing, and validating an efficient and straightforward electroanalytical protocol for determining the selected calcium channel blocker—amlodipine—using 3D-printed electrodes is demonstrated.

## Results and Discussion

### The Effect of Air Plasma Activation on the 3D-Printed Electrode’s Surface Properties

The effect of the plasma activation time on the 3D-printed electrode surface properties was analyzed by SEM (Figure 1). Figure 1a captures a significant difference between the nonplasma-treated (left) and plasma-treated surface (right). Kapton tape, which resists plasma discharge, was used to limit the surface. The image confirms the efficiency and precision of the activation allocation. The surface structure caused by the print head’s filament deposition trajectory is also observable in the image. The size of the present carbon nanoparticles (CB) ranges up to 100 nm (Figure 1b). The surface of the untreated electrode is covered with protruding parts of CB particles in the PLA matrix (Figure 1-ref). After only 5 s of plasma treatment, the surface structure changed. Plasma treatment causes the etching of the upper layers of PLA. Etching and an increase in PLA surface roughness have also been observed by AFM in previous works that studied the effect of atmospheric air plasma.^43,44^ Plasma etching of the PLA surface in air can be attributed to the disruption of polymer chains and the formation of oligomers. In addition, the removal of volatile products also occurs.^44^ Moreover, if the humidity of the supply air is not controlled and dry air is not used, then PLA hydrolysis (saponification) may also impact the etching of the PLA surface, mostly with prolonged plasma duration. PLA etching occurs in oxygen-containing discharges and has been observed in various atmospheres, such as Ar or CO_2_.^45,46^ PLA was etched locally at shorter plasma exposure times (up to 10 s). Full-surface etching occurs with a longer exposure time when plasma penetrates deeper, exposing more carbon-black surfaces. We observed relatively extensive removal of the polymer matrix for a longer plasma treatment duration, specifically at 160 s. Longer activations than 160 s caused the removal of large parts of PLA with CB, which led to free areas in the structure, causing intensive fouling of the plasma surface and reducing its effectiveness.

**Figure 1:**
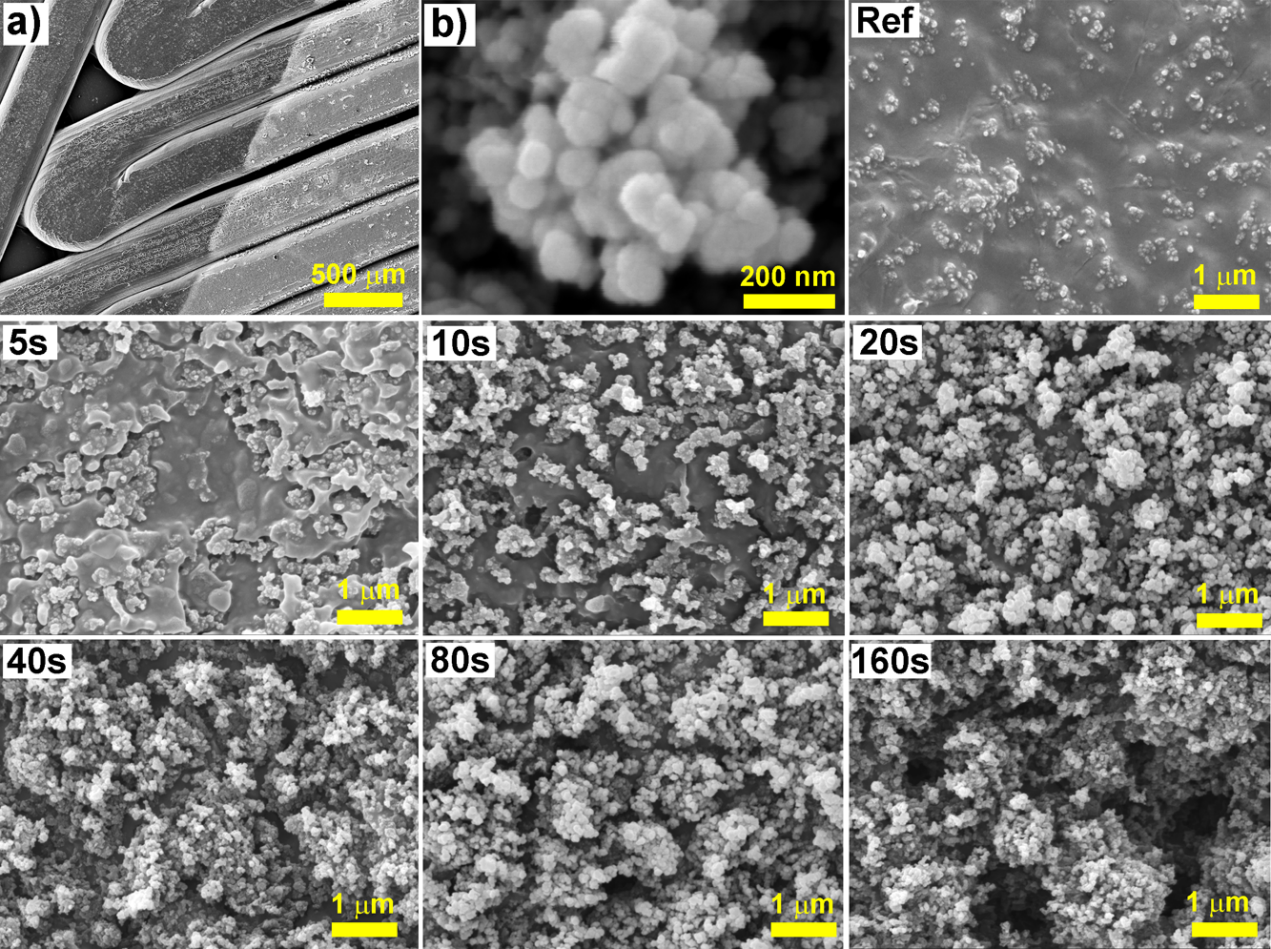
SEM measurements (a) interface of nonactivated and plasma-activated surface; (b) clusters of carbon nanoparticles; 3D-printed electrodes activated by low-temperature air plasma in duration from 0 s (ref) to 160 s.

The morphology of the electrode surface by using the SEM technique was also studied for chemically activated electrodes (Figure 2). Several solvents have been studied for chemical activation, especially DMF, acetone, acetonitrile, NaOH, etc.^47–49^ In this work, plasma-activated 3D-printed electrodes were compared with those activated by DMF and NaOH. The activation by NaOH requires the longest time for PLA to be successfully etched from the top layers, and the conductive CB particles become exposed. NaOH treatment lasting longer than 1 h did not lead to sufficient surface changes. Compared with NaOH, DMF activation requires shorter times, comparable to plasma treatment. Samples subjected to DMF activation had a specific structure. DMF treatment leaves PLA formations on the surface, and the duration of solvent exposure affects the extent of the polymer. The 60 s DMF activation appears to be more pronounced, with the effect of etching to a greater depth. Paradoxically, CB particles are not visible, even though other analysis methodologies indicate the availability of conductive particles. The morphology of the surface subjected to plasma treatment is comparable to that of NaOH activation, but the most significant difference is in the duration of activation. Compared to NaOH, the plasma causes finer, more detailed etching of PLA around the CB particles. The negative of the more extended plasma activation is that PLA is eliminated with carbon particles, leaving a free space on the electrode surface.

**Figure 2:**
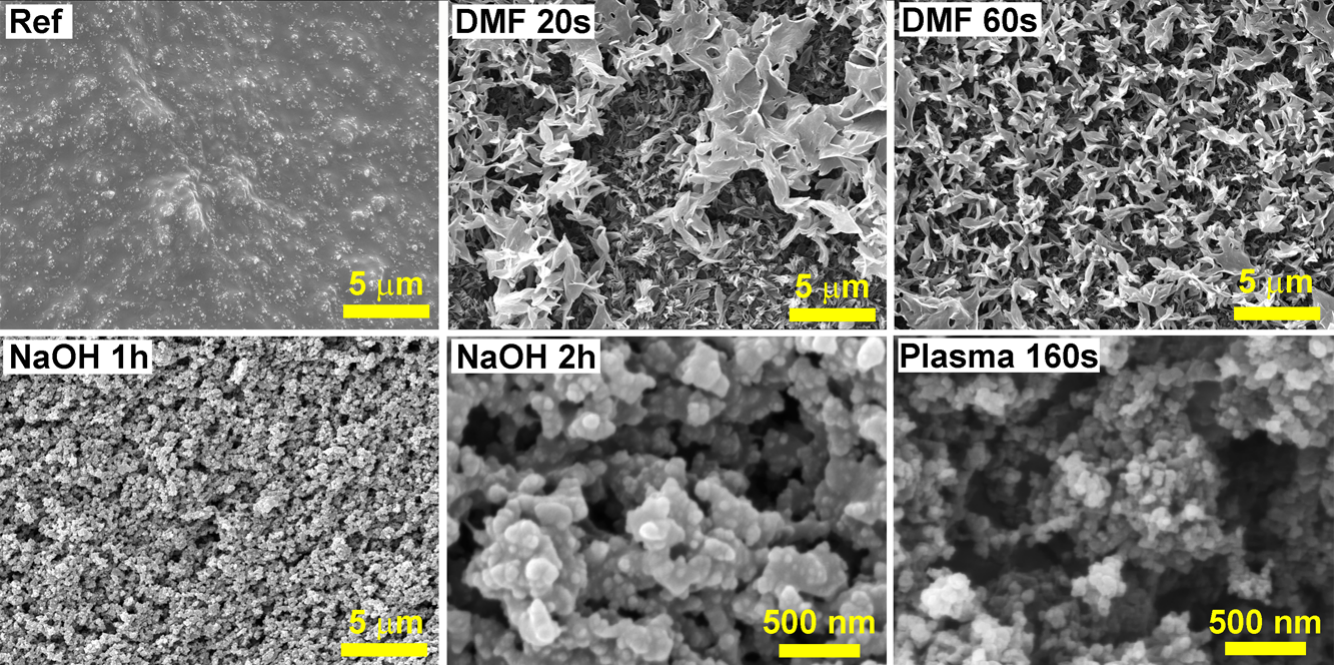
SEM images of chemically activated 3D-printed electrodes: (ref) before activation; activated by NaOH (1 and 2 h); *N,N*-dimethylformamide (DMF from 20 to 60 s); more detailed comparison of 160 s plasma and 2h of NaOH activated surface.

### Spectroscopic Measurements (Raman and XPS)

Carbon materials can be characterized in Raman spectra by D and G bands located at 1350 and 1580 cm^–1^. The G band is the result of sp^2^ in-plane carbon atom bond vibrations (graphitic crystal structure), while the D band is due to out-of-plane vibrations (sp^3^ hybridization) attributed to functionalization and structural defects.^50^ The intensity ratio of the D/G bands (*I*_D_/*I*_G_) indicates the level of defects in the material or the degree of carbon functionalization (oxidation). A low ratio value is typical for graphite and graphene; a high ratio value is characteristic for, e.g., graphene oxide, CB, biochar, etc.^51^ Figure 3a shows the spectra of the PLA standard in the nonplasma PLA/CB sample, and to compare the two electrode samples, plasma 10 and 160 s are shown. In the case of composite samples, only the D and G bands of CB were registered, with the absence of the characteristic bands for PLA. The dependence of the signal intensity ratio of the *I*_D_/*I*_G_ bands on the time of plasma treatment of the electrode surface has an increasing trend (Figure 3b). The *I*_D_/*I*_G_ ratio for the nonplasma sample increased from 1.01 to 1.04 after 5 s plasma treatment, and with more prolonged plasma activation, changed minimally to the highest value of 1.05. Thus, even more prolonged plasma treatment had a minimal effect on the formation of new defects in the CB structure. Due to the plasma treatment, an increase in the background (fluorescence) of the spectra was recorded (Figure 3). This phenomenon can be attributed to the degradation of PLA in the presence of oxygen, which was also observed during the laser etching of PLA in an O_2_ environment in the work of Glowacki.^52^ In the environment of inert gas He, this phenomenon no longer occurred, which the authors attribute to the slower PLA etching process.

**Figure 3:**
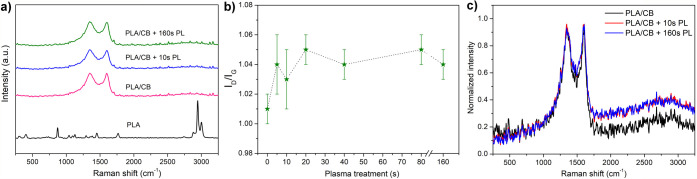
(a) Raman spectra of PLA and 3D-printed electrodes before and after plasma treatment; (b) dependency of the *I*_D_/*I*_G_ ratio on the time of plasma treatment; (c) effect of plasma treatment of the 3D-printed electrodes on the fluorescence increase.

The XPS analysis of the reference (PLA/CB) 3D-printed PLA/CB electrode contained main contributions from C and O elements, a small contribution of N, and debris from Si and Na ascribed to sample surface contamination. The XPS analysis of all plasma-treated samples revealed only contributions from C, N, and O elements, highlighting fast (5 s) cleaning of the PLA/CB surface from Na and Si contaminants. Table 1 summarizes the C, O, and N concentrations on the surface as determined by XPS analysis. With prolonged plasma treatment, the carbon-to-oxygen ratio (C/O) gradually decreased, corresponding to lower carbon content and increased oxygen contribution (Table 1). This was ascribed to surface functionalization by oxygen-containing species, as discussed further. On the other hand, the concentration of N after exposure to plasma slightly decreased for all treated samples, documenting that functionalization by nitrogen species did not occur.

**Table 1:** Elemental Composition of the Samples Corresponding to the C 1s, O 1s, and N 1s Peak Areas in XPS Spectra (in at %)

	C (at %)	O (at %)	N (at %)	C:O
PLA/CB	71.4	24.8	3.7	2.9
5 s	74.1	24.8	1.1	3.0
10 s	71.2	27.2	1.6	2.6
20 s	70.2	28.6	1.2	2.4
40 s	68.0	30.3	1.7	2.2
80 s	68.4	29.4	2.3	2.3
160 s	66.1	32.3	1.6	2.0

To discuss the evolution of the surface chemistry, the core-level C 1s and O 1s spectra are presented in Figure 4a,b. The XPS spectrum of PLA has already been elucidated in previous works.^53,54^ The XPS spectrum of untreated PLA/CB is formed by the following contributions. The main peak of the C 1s spectrum at 285.0 eV of binding energy corresponded to the signal from carbon in the methyl groups (C–C bonds). The signal around 287.0 eV is ascribed to carbon bonded in the backbone of PLA by C–O bonds, and the signal positioned around 289.1 eV belongs to carbon bonded in the polymer by the O=C–O bonds. The O 1s spectrum documents the presence of C=O bonds (at 532.3 eV) and C–O bonds (at 533.5 eV) detected in the C 1s spectrum. Notably, the components in both C 1s and O 1s overlap with the signal from adventitious carbon or hydrocarbon contaminants arising from exposure of the materials to ambient air. Similarly, the contribution from carbon nanoparticles detected on the surface by SEM overlaps with that of the detected spectra.

**Figure 4:**
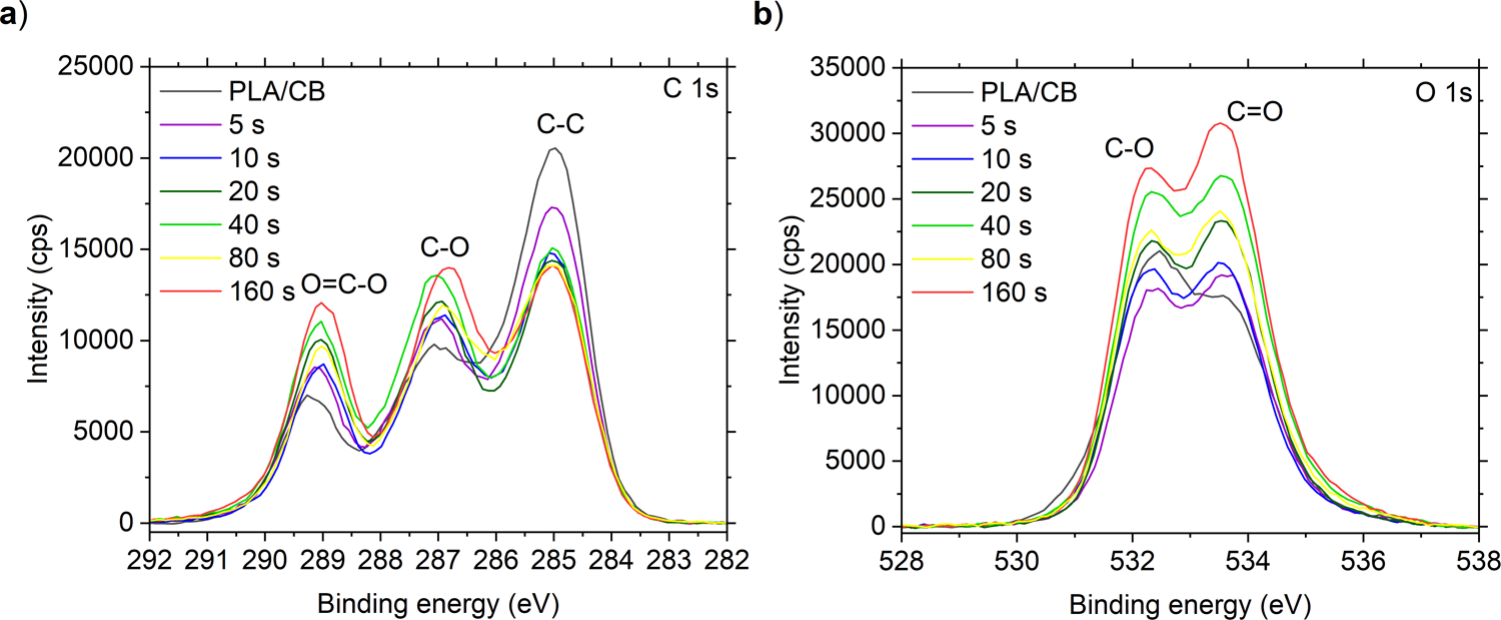
Core-level XPS spectra of 3D-printed PLA/CB electrode and electrodes treated by plasma for 5–160 s. The C 1s spectrum (a) and the O 1s spectrum (b).

The plasma treatment led to pronounced changes in the surface chemistry already after 5 s, and the surface modification was further enhanced with the prolonged treatment time. The intensity of the C–C peak decreased, corresponding to the cleavage of methyl groups in the polymer structure and cleaning of adventitious carbon/hydrocarbon contaminants. This process was followed by functionalization with alcohol (C–OH) and ketone (C=O) groups, leading to increased intensity of the corresponding signals in C 1s and O 1s spectra. The detected effect of surface modification by DCSBD treatment is similar to that reported in related works.^55,56^ The maximum concentration of C–O and O=C–O groups was detected after the longest treatment (160 s).

### Measurement of Surface-Free Energy by the Contact Angle Method

The wetting contact angle method was used to characterize the change in surface-free energy (as a sum of its polar (σ_p_) and dispersive (σ_d_) components) and the wettability of electrodes subjected to plasma surface treatment. The polar surface energy component corresponds to acid–base and dipole–dipole molecular interactions, enabling charge transport between molecules, improving electrode wettability, and thus providing better interaction between the target analyte and electrode surface. The dispersive component of the surface energy is related to the much weaker interaction between the electron clouds of molecules on the surface. Results in Figure 5a, evaluated by the Owens–Wendt method, show a gradual increase of total surface energy (σ_tot_) with plasma treatment time from the low initial value of 35.55 mJ·m^–2^ for the reference electrode to a maximum of 66.4 mJ·m^–2^ evaluated for 80 s plasma-treated electrodes, respectively. Due to surface oxidation (in accordance with XPS results) and removal of hydrophobic PLA by plasma (in accordance with SEM results), the polar component increases from 29.5 mJ·m^–2^ (nonactivated electrodes) to a maximum of 65.4 mJ·m^–2^ (80 s plasma activated). Subsequently, the dispersive component decreases with plasma treatment time from 6.1 mJ·m^–2^ (reference sample) to 3.3 mJ·m^–2^ after 5 s of plasma activation and reaches minimum values of 0.8 mJ·m^–2^ for plasma activation longer than 10 s. In the case of chemical activation using DMF and NaOH, significant increases in the dispersion component of the surface energy were recorded (Figure 5b). This can be attributed to the exposure of CB nanoparticles, which have a hydrophobic character. The exception was a 2 h activation with NaOH, resulting in a higher polar than dispersive component. It can be related to the removal of larger CB blocks due to intensive binder-PLA removal. Although both electrode materials, PLA and CB, have a hydrophobic nature, the much larger specific surface area of CB enhances the intensity of the dispersion component. Therefore, with CB removal, the ratio between surface-free energy components has changed in favor of the polar component.

**Figure 5:**
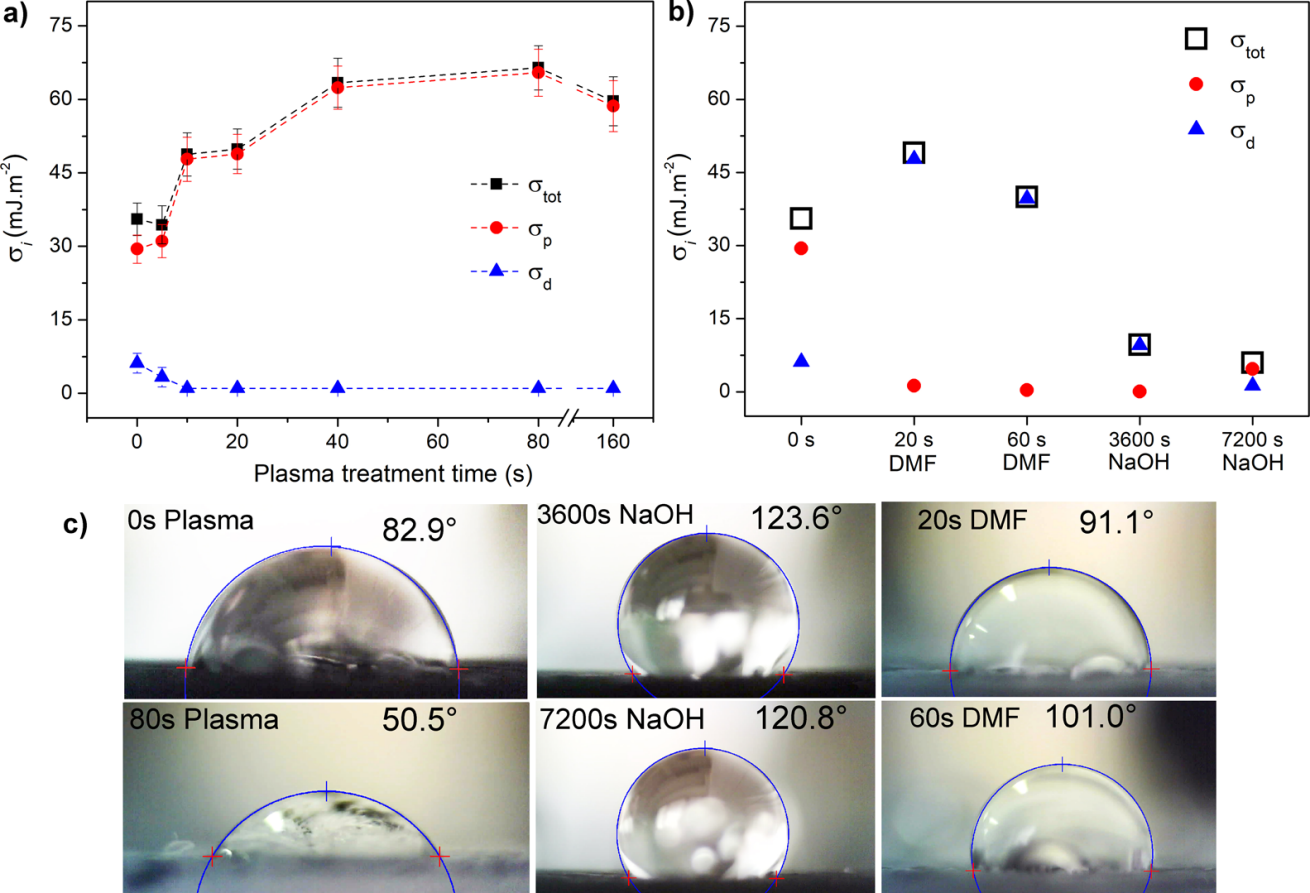
Dependency of 3D-printed PLA/CB electrodes surface-free energy (σ_tot_), polar (σ_p_), and dispersive (σ_d_) components of surface energy on plasma (a) and chemical (b) treatment time. (c) Images of water contact angle measurements (WCA).

The better wettability of the plasma-activated electrodes than chemically activated electrodes can be shown by the decrease in water contact angle (WCA) (Figure 5c). Before plasma activation, the electrodes exhibited a WCA of 85 ± 3°, which is on the edge of distinguishing between the hydrophilic (<90°) and hydrophobic (>90°) nature of the surface. These values are in line with other authors’ measurements, in which they examined various commercial PLA/carbon filaments and achieved WCA in the range of 73–86°.^49^ This result indicates that before activation, the surface of the electrodes and CB particles are wholly covered with PLA. After activation, removing hydrophobic PLA and also PLA oxidation, by plasma treatment led to the exposure of the plasma-oxidized CB with more hydrophilic character than nonoxidized CB.^57,58^ Thus, the WCA decreased with plasma treatment time and reached a minimum value of 54 ± 4° for plasma activation longer than 80 s. On the other hand, chemical activation led to an increase in the contact angle and a more pronounced hydrophobic nature of the electrode surface, which could be related to the measured higher proportion of the dispersive component of the surface energy than in the case of the reference and plasma-activated electrodes. Plasma activation thus proves to be more suitable for increasing the hydrophilicity of the electrode surface, which could be beneficial for electrochemical measurements.

### Electrochemical Characterization

Electrochemical analysis was performed using cyclic voltammetry (CV). The background current (blank) measurement was carried out in 0.1 M KCl for electrodes activated by plasma treatment and chemical activation using DMF and 1 M NaOH (Figure 6). The respective CV profiles of 3D-printed electrodes activated by plasma (Figure 6a) recorded an increase in the intensity of the capacitive current with the prolongation of the plasma treatment up to 80 s activations. This could be associated with the exposure of CB particles, which provide their typical surface properties. Besides, a longer plasma treatment time did not alter the background current. As to nonactivated electrodes, the results showed no significant electrochemical activity, which is consistent with the results of previous measurements. Herein, it should also be emphasized that the increasing background current may be undesirable for sensitive and reliable detection in electrochemical analysis. CV records of chemical activation using 1 M NaOH indicated a negligible change in the intensity of the capacitive current with a duration of activation from 1 to 2 h (Figure 6b). In the case of DMF, the alteration in the capacitive current concerning the activation time (for 20 and 60 s) was negligible (Figure 6c). Compared to chemically activated electrodes, plasma-treated electrodes achieved comparable capacitive currents when exposed to plasma within 20 s.

**Figure 6:**
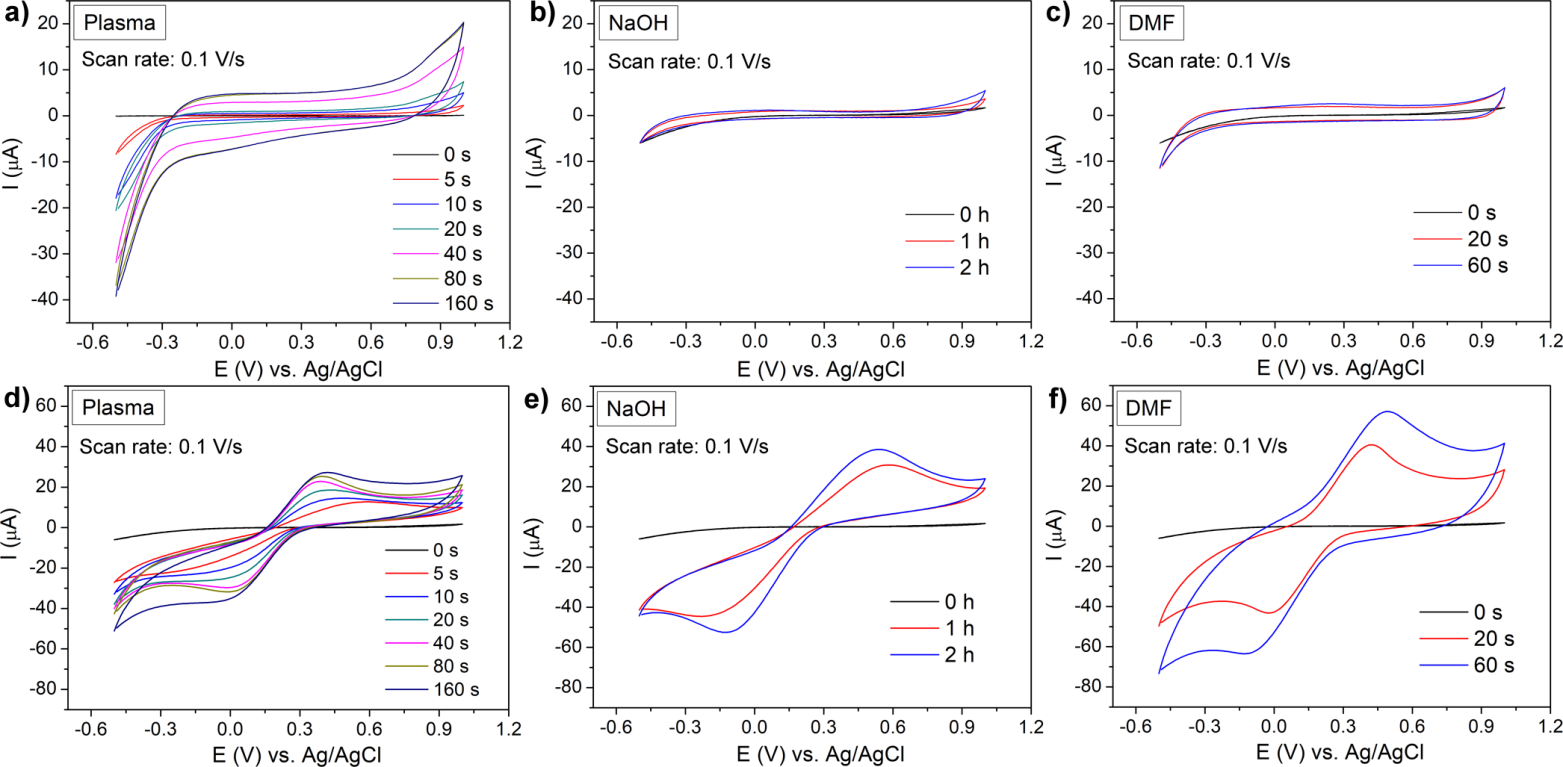
CV records of differently activated 3D-printed electrodes measured at a scan rate of 0.1 V·s^–1^. (a–c) Background cyclic voltammograms in 0.1 M KCl and (d–f) CV records in 0.1 M KCl containing 1 mM [Fe­(CN)_6_]^3-/4-^: (a, d) plasma activation, (b, e) 1 M NaOH, and (c, f) DMF.

By measuring the CV in 0.1 M KCl containing redox indicator 1 mM [Fe­(CN)_6_]^3–/4–^ by applying a potential window from −0.5 to +1.0 V at a scan rate of 0.1 V·s^–1^, the peak currents and the separation potential were assessed (Figure 6). The values of measured currents (after background correction), peak-to-peak separation potential, and other redox characteristics are listed in Table 2. As evidenced, the oxidation and reduction peak currents of the redox indicator increase with increasing plasma surface treatment time (Figure 7d). This trend could be explained by making conductive carbon nanoparticles accessible by partially removing PLA from the electrode surface. The electrode without surface activation did not provide any significant current response. In addition, we observed a weak electrochemical activity of the redox indicator for the electrode treated by plasma for 5 s. The peak-to-peak separation potential (Δ*E*_p_) was evaluated to assess the reversibility of the one-electron transfer related to the electrode reaction of [Fe­(CN)_6_]^3–/4–^ (theoretical value of 0.059 V). All samples, regardless of the plasma activation time, reached a high Δ*E*_p_ in the range of 0.37–0.45 V. This phenomenon could be attributed to the mechanism of the ongoing redox reaction of the redox indicator related to the inner sphere electron transport, which is significantly affected by surface properties.^59^ The most favorable reversibility, with the lowest Δ*E*_p_ of 0.37–0.38 V, was evaluated for the electrodes treated by plasma for 40 and 80 s. Figure S1 shows the current dependence on the square root of the scan rate for plasma, NaOH (1 h), and DMF (20 s) activation, respectively. The highest Δ*E*_p_ value of 0.45 V, assessed for the most extended plasma treatment, can be caused by the more oxidized nature of the electrode surface. In this case, the highly oxidized surface with partial negative charge repels [Fe­(CN)_6_]^3–/4–^, slowing charge transfer, impairing kinetics, and causing greater peak separation. Considering the achieved redox parameters and the practical advantages of a shorter plasma time, 40 s was considered the most suitable plasma activation time.

**Table 2:** Values of the Maximum Current and Potential of Oxidation and Reduction Peaks, *I*_pa_/*I*_pc_ Ratio, Separation Potential (Δ*E*_p_), Electrochemical Electrode Area (*A*), and Charge-Transfer Resistance (*R*_ct_) for Individual Activation Methods of 3D-Printed Electrodes

*t* (s)	*I*_pa_ (μA)	*I*_pc_ (μA)	*I*_pa_/*I*_pc_	*E*_pa_ (V)	*E*_pc_ (V)	Δ*E*_p_ (V)	*A* (cm^2^)	*R*_ct_ (Ω·cm^2^)
0	×	×	×	×	×	×	×	1423 ± 151
Air Plasma								
5	4.43	×	×	0.46	×	×	0.218 ± 0.004	190 ± 15
10	10.25	–18.40	–0.56	0.39	–0.02	0.41	0.260 ± 0.040	165 ± 7
20	15.47	–22.99	–0.67	0.38	–0.03	0.41	0.567 ± 0.090	144 ± 6
40	22.25	–27.61	–0.81	0.37	0.00	0.37	0.794 ± 0.068	110 ± 5
80	23.31	–28.99	–0.80	0.38	0.00	0.38	0.889 ± 0.014	114 ± 4
160	20.72	–31.13	–0.67	0.39	–0.06	0.45	0.909 ± 0.044	120 ± 6
NaOH								
3600	16.53	–36.01	–0.46	0.51	–0.20	0.71	0.376 ± 0.011	×
7200	30.38	–47.69	–0.64	0.50	–0.11	0.61	×	×
DMF								
20	27.71	–35.78	–0.77	0.41	–0.02	0.43	0.491 ± 0.062	125 ± 8
60	26.85	–47.94	–0.56	0.46	–0.11	0.57	×	×

**Figure 7:**
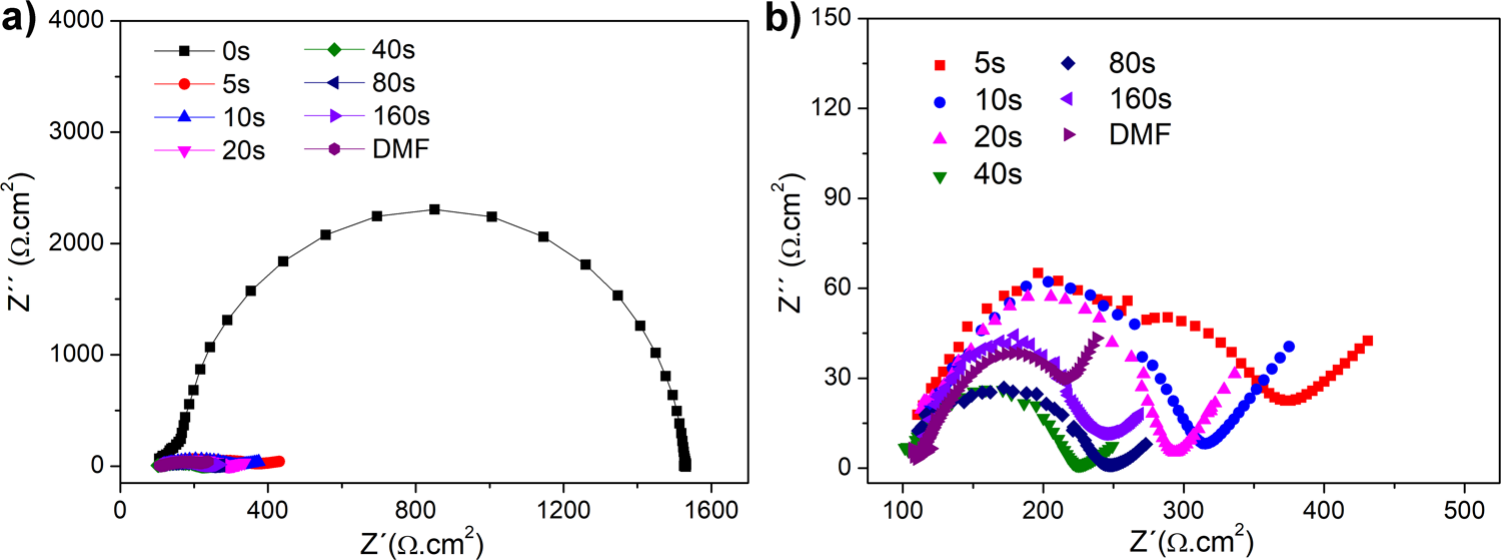
EIS measurement in 0.1 M KCl containing 1 mM [Fe­(CN)_6_]^3–/4–^ of reference 3D-printed electrodes and with plasma and DMF-activated electrodes (a). The zoomed-in area was measured for activated electrodes (b).

CV measurement under the same conditions as for plasma-treated electrodes was also undertaken for the chemical activation methods. Chemical activation was realized for the NaOH solvent with a fixed concentration of 1 M (Figure 6e). In this case, a significantly longer activation time is required to improve the redox properties of the redox indicator on electrodes compared to the plasma or DMF activation. At times shorter than 1 h, no relevant current responses of the redox indicator were observed, and it was impossible to reliably evaluate the oxidation and reduction peak potentials. Extending the activation time to 2 h resulted in higher electrochemical activity, and Δ*E*_p_ was reduced from 0.71 to 0.61 V (Table 2). However, Δ*E*_p_ was still higher than in the case of 40 s plasma activation and DMF activation. In the case of DMF, a longer 60 s activation improved the recorded current response of the redox indicator on 3D-printed electrodes. Moreover, compared to 20 s activation, the reaction kinetics slowed down, and the Δ*E*_p_ got higher (Figure 6f). For DMF, the short activation times observed were unexpected, as other authors have reported that at least 10 min is considered the appropriate activation time.^60,61^

The electrochemical response of the activated electrodes in 0.1 M KCl containing 1 mM [Fe­(CN)_6_]^3–/4^^–^ at various scan rates (from 0.005 to 0.5 V·s^–1^) was used to calculate the active surface area. Considering the previous electrochemical measurements and the practical aspect of the duration of the activation process, DMF (20 s) and NaOH (1 h) were chosen to compare chemical activation with plasma activation for the active area evaluation. Figure S1 shows the current dependence on the square root of the scan rate for plasma, NaOH (1 h), and DMF (20 s) activation, respectively. Higher scan rate values reduce the diffusion layer, resulting in a higher signal response of the peak current.^5^ Enabling conductive carbon nanoparticles accessible by the activation process increased the size of the electroactive surface. In all cases, the linear dependence of the peak current on the square root of the scan rate indicated a diffusion-driven reduction process of [Fe­(CN)_6_]^3–/4–^. Chemical activation processes also resulted in the same results. The active surface area was calculated using the Randles–Sevcik Equation (eq 1)­

$$i_{p,\mathrm{ox}}=2.686\times 10^{5}n^{3/2}AD^{1/2}cv^{1/2}$$ 1

where *n* is the number of electrons transferred in the electrochemical process, *A* the obtained electroactive area (cm^2^), *D* the diffusion coefficient of the redox probe (7.6 × 10^–6^ cm^2^ s^–1^), *c* the concentration of the redox probe (mol·mL^–1^), and *v* the applied voltammetric scan rate (V·s^–1^). With the prolonged plasma treatment, the electrode surface area increased, extending the activation time from 5 to 160 s, from 0.22 to 0.91 cm^2^. Regarding the chemically activated electrodes using DMF (20 s), they provided a higher surface area than NaOH (1 h), namely, 0.49 versus 0.38 cm^2^. However, these values are lower than those achieved in the case of plasma activation for a time 20 s or longer. These results are surprising because in the case of chemical activation, the entire electrode heads were immersed in solvents; thus, the sides of the electrodes were also activated. This does not apply to plasma activation, in which only the front and back of the electrode heads were activated, but not their sides. However, results indicated that plasma activation with the same treatment time as DMF (20 s) was more effective in etching PLA and exposing conductive and electroactive CB nanoparticles.

### Electrochemical Impedance Spectroscopy

EIS spectra (Figure 7) were recorded in the 1 Hz to 100 kHz frequency range at an amplitude of 0.2 V. The charge transfer resistance (*R*_ct_) values were evaluated using the alternate electrical equivalent circuits (*R*[*R*]/*Q*) for nonactivated electrodes (*R*[RW]/*Q*) for plasma- and DMF-activated electrodes, as shown in Table 2. Evidently, nonactivated electrodes showed the highest *R*_ct_ of 1423 Ω·cm^2^. Even with the shortest plasma exposure of 5 s, there was a significant decrease in *R*_ct_ to 190 Ω·cm^2^. With 10 and 20 s plasma exposure, a decrease in *R*_ct_ from 165 to 144 Ω·cm^2^ was noticed. *R*_ct_ values changed slightly during plasma activation longer than 40 s. At 40, 80, and 160 s, *R*_ct_ reached 110, 114, and 120 Ω·cm^2^, respectively. Electrodes activated with DMF for 20 s achieved a slightly lower *R*_ct_ of 125 Ω·cm^2^. For comparison, Pereira et al.^46^ achieved *R*_ct_ of 394 and 1915 Ω·cm^2^ using plasma discharge in O_2_ and CO_2_ atmosphere, respectively.

### Comparison of Activation Processes of 3D-Printed PLA/CB Electrodes

In this section, we aim to compare various activation procedures employed by other authors with those in our work. All of the compared works used Protopasta’s (Protoplant Inc., Vancouver) Composite Conductive PLA filament to prepare 3D-printed sensors. Table 3 presents the comparative attributes of individual activation procedures, including activation conditions, activation time, and selected electrochemical parameters Δ*E*_p_ and *R*_ct_. In all works, an electrolyte based on a [Fe­(CN)_6_]^3–/4–^ redox mediator was used. As of today, various methods exist for removing excess PLA and subsequently activating the surface of a 3D-printed PLA/CB electrode. These include physical, chemical, electrochemical, and mechanical activation techniques. In the study conducted by Pereira et al.,^46^ plasma treatment was employed for surface modification in different gaseous environments, specifically O_2_ and CO_2_. Compared to our methodology, the plasma treatment duration was longer—up to 120 s—and it operates in a closed chamber. For both gases, the resulting values of Δ*E*_p_ were lower and *R*_ct_ were higher, with a particularly notable difference in the case of O_2_. Farines et al.^62^ applied plasma modification using a nonthermal Ar plasma jet with a treatment duration of 5 min. Compared to our plasma-activated electrodes, they achieved a much higher *R*_ct_ of 6800 Ω and Δ*E*_p_ of 0.660 V. Further research^63^ in the domain of plasma treatment utilized an air plasma jet pen, with a modification time of 2 min. The electrochemical results demonstrated enhanced electrochemical activity, with Δ*E*_p_ = 0.144 V and *R*_ct_ = 104 Ω, due to the exposure of conductive carbon particles following the removal of insulating PLA. Although the activation time was three times longer than in our work, the electrochemical parameters were significantly better. From an application perspective, the use of the air plasma jet pen appears to be the most promising among plasma modifications. The study by Kozłowska et al.^64^ focused on the effects of microwave activation on PLA/CB electrodes. After 15 min of microwave exposure, the electrodes exhibited Δ*E*_p_ values of 0.420 V and *R*_ct_ of 1100 Ω·cm^2^.

**Table 3:** Comparison of Different Activation Processes of 3D-Printed PLA/CB Sensors Made of Protopasta Conducting Filament on Their Duration and Electrochemical Values of Separation Potential (Δ*E*_p_) and Charge Transfer Resistance (*R*_ct_) Measured in the [Fe­(CN)_6_]^3–/4–^ Redox Mediator

filament	redox probe	activation	duration	Δ*E*_p_ (V)	*R*_ct_	ref
PLA/CB Protopasta	1 mM [Fe(CN)_6_]^3–/4–^ in 0.1 M KCl	DCSBD plasma	40 s	0.37	117 Ω·cm^2^	this work
		DMF	20 s	0.43	115 Ω·cm^2^	
	1 mM [Fe(CN)_6_]^3–/4–^ in 0.1 M KCl	plasma O_2_	2 min	0.156	394 Ω·cm^2^	^46^
		plasma CO_2_		0.301	1915 Ω·cm^2^	
	5 mM [Fe(CN)_6_]^3–/4–^ in 0.1 M KCl	Ar plasma jet	5 min	0.66	6800 Ω	^62^
	1 mM [Fe(CN)_6_]^3–/4–^ in 0.01 M PB	microwave 70 °C, 100 W	15 min	0.42	1100 Ω·cm^2^	^64^
	1 mM [Fe(CN)_6_]^3–/4–^ in 0.1 M KCl	laser 280 mW	200 s	0.136	1410 Ω	^65^
		electrochem. 0.5 M NaOH				
	1 mM [Fe(CN)_6_]^3–/4–^ in 0.5 M H_2_SO_4_	mechanical sandpaper	∼34 min	0.092	170 Ω·cm^2^	^66^
		30 min NaOH, ultrasonic bath				
		electrochem. 0.5 M NaOH				
	1 mM [Fe(CN)_6_]^3–/4–^ in 0.1 M KCl	mechanical	400 s	0.297	×	^67^
		electrochem. 0.5 M NaOH				
	1 mM [Fe(CN)_6_]^3–/4–^ in 0.1 M KCl	mechanical	×	0.19	120 Ω	^35^
		CO_2_ laser				
		electrochem. 0.5 M NaOH				
	2 mM [Fe(CN)_6_]^3–/4–^ in 1 M KCl	air plasma	2 min	0.144	104 Ω	^63^
		jet pen				

Several studies have investigated the use of the combined modification techniques. In general, combining multiple activation methods enhances the electrochemical parameters of 3D-printed PLA/CB electrodes, albeit at the expense of increased time and process complexity. Typically, mechanical polishing using sandpaper is performed as the initial step to activate the electrode surface. Rocha et al.^65^ subsequently subjected the electrodes to electrochemical treatment in NaOH, achieving a Δ*E*_p_ value of 0.297 V. A similar approach was adopted by Rodrigues et al.,^66^ who inserted an additional sonication step in 0.5 M NaOH for 30 min between the mechanical and electrochemical activation stages. This approach yielded enhanced electrochemical performance with a very low Δ*E*_p_ of 0.092 V and an *R*_ct_ of 170 Ω·cm^2^. Instead of sonication, Veloso et al.^67^ employed CO_2_ laser treatment as the second step, resulting in an even lower *R*_ct_ but higher Δ*E*_p_ of 0.190 V. Slightly enhanced Δ*E*_p_ but much higher *R*_ct_ were achieved by Carvalho et al.^35^ when omitting the initial mechanical surface treatment.

As can be concluded, the results of our work demonstrate the advantages of air plasma, particularly in its very short activation process, use of ambient air, low temperature, low cost, and absence of toxic liquids. Moreover, by using our DCSBD plasma in an ambient atmosphere, we achieved comparable electrochemical results between single-step processes. However, the best electrochemical results were achieved in the work of Siquera et al.,^63^ who used a straightforward activation method involving a 2-min plasma jet pen in ambient air. As shown by other authors, further improvement of electrochemical activity can be achieved by combining multiple activation processes, but at the cost of longer duration and more complex processes.

### Application of 3D-Printed Electrodes in Drug Analysis

In order to demonstrate the practical applicability of 3D-printed electrodes (activated by air plasma for 40 s), a novel, simple, and rapid electroanalytical protocol in drug analysis was established. Based on the above electrochemical results, only samples activated by 40 s of plasma were selected for this analysis. For this purpose, amlodipine (AML), a calcium channel blocker widely used in hypertension treatment, was considered to be the target analyte. This drug is commonly used to manage high blood pressure. However, their excessive concentration can lead to serious side effects, highlighting the need for reliable analytical methods.

The initial step involved studying the electrochemical behavior of 1 mM AML in Britton–Robinson (BR) buffer solutions with pH values ranging from 2 to 12 on a 3D-printed electrode using CV. The results of this section, together with the proposal for the mechanism of electrochemical oxidation of AML, are presented and discussed in the Supporting Information (Figure S2). The BR buffer at pH 6 was chosen as the supporting electrolyte for the subsequent measurements. Additionally, to demonstrate the usefulness of plasma activation, we compared the current responses of 1 mM AML in BR buffer at pH 6, obtained using CV on untreated (nonplasma) and 40s plasma-activated 3D-printed electrodes. In this respect, plasma treatment of the 3D-printed electrode substantially enhances its electrochemical performance, as evidenced by a more distinct and better-defined current response of AML, as well as a significantly lower background current observed in the CV record (Figure 8). The improved voltammetric profile of the analyte indicates that plasma activation effectively modifies the electrode surface, leading to enhanced electron transfer kinetics and a greater electroactive surface area. Plasma activation also removes impurities from the surface and increases its roughness, making more active sites available for the AML. In addition, it can introduce oxygen-containing functional groups (such as hydroxyl, carbonyl, or carboxyl groups) onto the electrode surface, which facilitates electron transfer and promotes better contact with the BR buffer and AML. These findings emphasize the importance and benefits of plasma activation as a surface modification strategy to enhance the performance of 3D-printed electrodes for the electrochemical sensing of AML.

**Figure 8:**
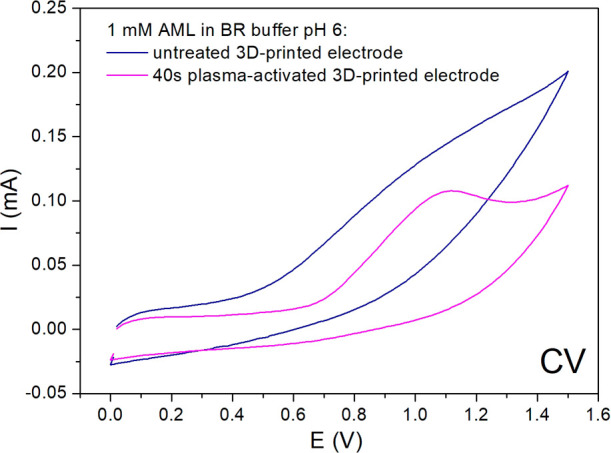
CV records of 1 mM AML in BR buffer at pH 6 obtained on nonactivated and 40 s plasma-activated 3D-printed electrodes, recorded at a scan rate of 100 mV·s^–1^.

The set instrumental parameters of quantification techniques such as differential pulse voltammetry (DPV) and square-wave voltammetry (SWV) were as follows: modulation amplitude of 100 mV, modulation time of 150 ms for DPV, and amplitude of 75 mV and frequency 10 Hz for SWV. These values represent the optimization process results, which are discussed in detail in the Supporting Information (Figures S3 and S4).

Calibration solutions of AML were prepared by adding specific volumes of 0.1 mM AML to a BR buffer of pH 6 to a final volume of 20 mL in the electrochemical cell. Measurements were performed using both SWV and DPV under optimized conditions, and before each, the plasma-activated 3D-printed electrode surface was cleaned by rinsing with deionized water. As a demonstrative example, Figure 9 shows the corresponding DP voltammograms obtained on the 40s plasma-activated 3D-printed electrode with a distinct oxidation peak of AML appearing at +0.7 V. This peak increased linearly with ascending AML concentration from 0.7 to 10 μM for DPV (from 0.5 to 30 μM for SWV, the results not shown). The particular calibration graph *I*_p_ = *f*(*c*_AML_) for DPV is given in the inset of Figure 9. By evaluating the obtained calibration graphs, essential analytical parameters for the determination of AML using DPV and SWV were assessed (Table 3).

**Figure 9:**
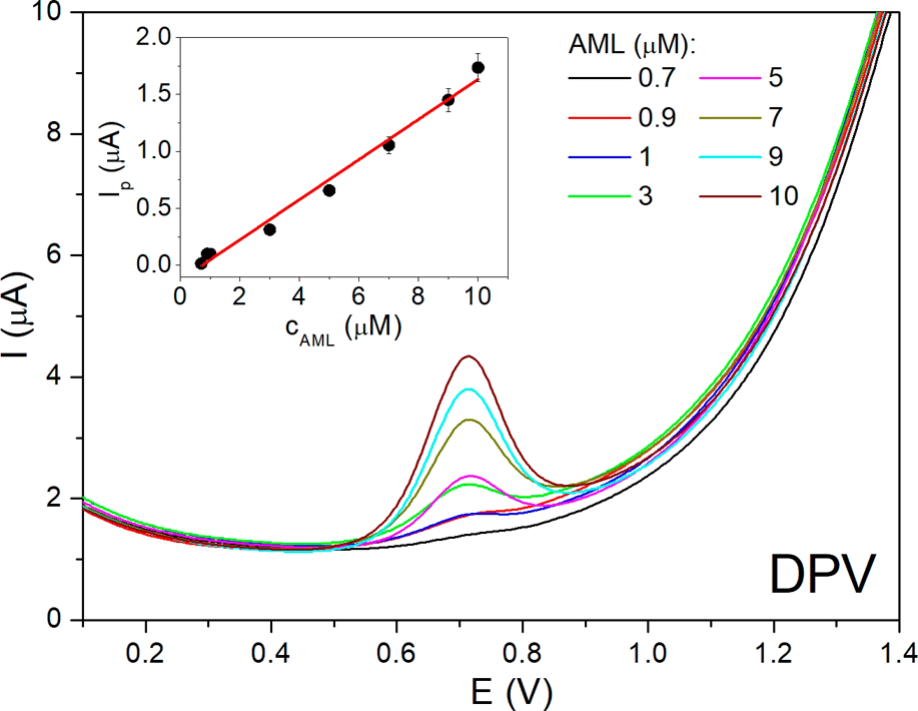
DP voltammograms for various concentrations of AML in BR buffer at pH 6 on 40 s plasma-activated 3D-printed electrode (optimized parameters: modulation amplitude, 100 mV; modulation time, 150 ms). Inset: calibration curve: *I*_p_ = *f*(*c*_AML_).

The analytical performance evaluation is presented in Table 4, demonstrating that DPV exhibits slightly higher sensitivity than SWV, as reflected by its higher slope value (0.176 μA/μM) compared to SWV (0.134 μA/μM) and achieves lower limit of detection (LOD) values. On the contrary, SWV provides a broader linear concentration range of 0.5–30 μM, whereas DPV is narrowed to 0.7–10 μM. Despite some differences, both DPV and SWV demonstrate acceptable performance for AML determination. Regarding the repeatability study, 20 consecutive measurements were carried out using a 9 μM AML in BR buffer at pH 6, performed without any working electrode cleaning between individual measurements. The resulting relative standard deviations (RSD) were 8.7 and 6.6% for SWV and DPV, respectively, indicating sufficient precision in the developed electroanalytical protocol using 40s plasma-activated 3D-printed electrodes. In this case, DPV indicates a steadier performance with a lower RSD (6.6%) than SWV (8.7%).

**Table 4:** Analytical Parameters for AML Determination on 40 s Plasma-Activated 3D-Printed Electrode

	selected voltammetric technique	
analytical parameter	SWV	DPV
intercept (μA)	–0.0088 ± 0.0075	–0.1268 ± 0.0055
slope (μA/μM)	0.134 ± 0.024	0.176 ± 0.046
linear concentration range (μM)	0.5–30	0.7–10
coefficient of determination *R* ^2^	0.997	0.986
LOD (μM)	0.17	0.09
repeatability (RSD %, 9 μM AML, *n* = 20)	8.7	6.6

The final part of the practical application of 40 s plasma-activated 3D-printed electrodes was focused on real sample analysis using the previously optimized instrumental parameters for both DPV and SWV. For this purpose, the pharmaceutical product Agen 5 from Zentiva (Czech Republic) was obtained from Ostrava Municipal Hospital (Czech Republic). The product contains AML as the active pharmaceutical ingredient in an amount of 5 mg per tablet. As for the sample preparation, ten tablets were weighed and finely ground into a white powder using an agate mortar to obtain a homogeneous sample. An amount equivalent to one tablet was precisely weighed on an analytical balance, dissolved in a small volume of methanol, and filtered to remove any insoluble components. The filtrate was quantitatively transferred into a 50 mL volumetric flask and diluted to the mark with methanol. For the following electrochemical determination of AML with DPV and SWV methods, 0.5 mL of the sample solution was pipetted into the electrochemical cell containing 20 mL of BR buffer of pH 6. The AML concentration was calculated using the standard addition method, involving adding defined volumes of a 1 mM AML standard solution (0.2–0.4 mL). Measurements were performed three times, and after each analysis, the 40 s plasma-activated 3D-electrode surface was only rinsed with deionized water. The DP voltammograms are demonstratively presented in Figure 10. As evidenced, successive additions of the standard led to a gradual and proportional increase in the oxidation peak of AML at +0.7 V, consistent with the ascending concentration levels of AML in the electrochemical cell. Regarding the fluctuation in background current for AML in the pharmaceutical sample and after applying the standard addition method, as observed in the illustrative example in Figure 10, this phenomenon is quite common in electroanalysis using the standard addition method. Herein, it can be attributed to slight variations in the 3D-printed electrode surface state caused by the adsorption of matrix components (such as excipients present in tablet samples) from the Agen 5 sample, which was applied first (after analysis of the blank solution). The subsequent standard additions of 1 mM AML typically reduced the matrix effect, resulting in a lower background current.

**Figure 10:**
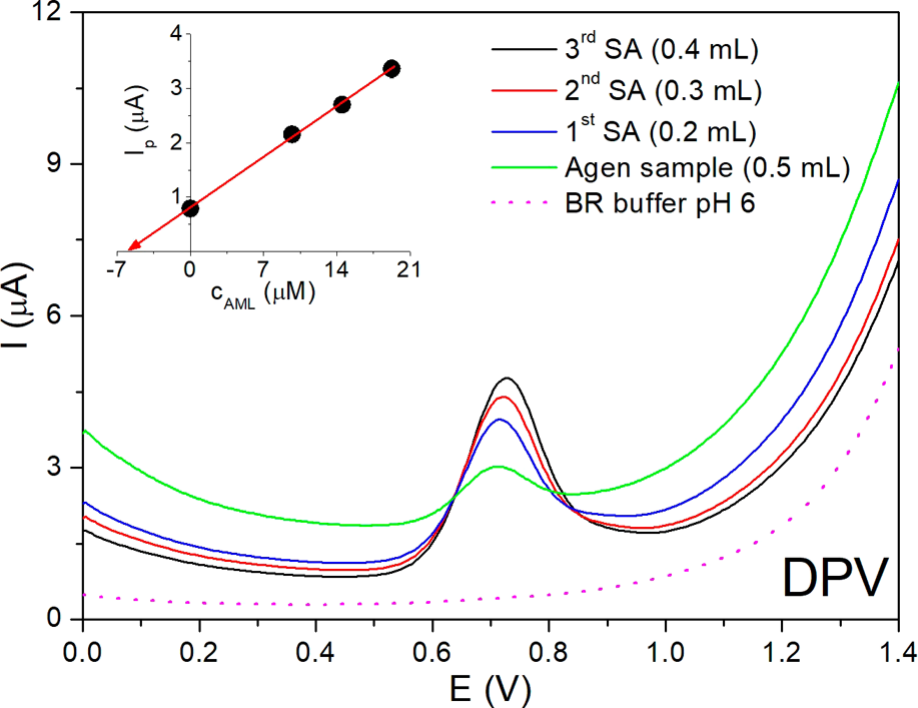
DP voltammograms of the analysis of the pharmaceutical product Agen 5 in BR buffer of pH 6 using a 40 s plasma-activated 3D-printed electrode with standard additions (SA) of 1 mM AML: 0.2, 0.3, and 0.4 mL. Inset: graphical representation of AML determination using the standard addition method.

Following the data evaluation, the concentration of AML within the electrochemical system was obtained through graphical analysis of the standard addition method (inset of Figure 10). Table 5 summarizes essential information obtained from the analysis, specifically the determined amount of AML using DPV and SWV, calculated as the mean (*x̅*) of three replicate measurements, along with the 95% confidence interval and the recovery values. After application of the spike-recovery assay by fortifying the pharmaceutical sample with 1 mg of AML standard solution, the obtained recovery values of 97.1 and 105.4% for SWV and DPV, respectively, confirm the favorable accuracy and reliability of the developed electroanalytical protocol utilizing 40s plasma-activated 3D-printed electrodes. Furthermore, the results clearly reveal the potential of rapid atmospheric air plasma activation of 3D-printed electrodes for future applications in routine pharmaceutical and clinical analyses.

**Table 5:** AML Amount Determined by the Standard Addition Method with Spike-Recovery Assay in Pharmaceutical Product Agen via an Optimized Electroanalytical Protocol (*n* = 3)

		selected voltammetric technique							
		SWV				DPV			
pharmaceutical product	label (mg)	found (mg)	fortified (mg)	found after spiking (mg)	recovery (%)	found (mg)	fortified (mg)	found after spiking (mg)	recovery (%)
Agen 5	5	4.85 ± 0.61	1.0	5.83 ± 0.28	97.1	5.27 ± 0.50	1.0	6.32 ± 0.28	105.4

Table S1 summarizes the comparison of the analytical performance of the proposed method with selected voltammetric methods for the determination of AML reported between 2021 and 2025. The plasma-activated 3D-printed electrodes show comparable or superior performance in terms of LOD and simplicity of preparation while also offering advantages such as rapid fabrication, environmental sustainability, and cost-effectiveness.

## Conclusions

This study demonstrated that atmospheric air plasma treatment offers a rapid, efficient, and environmentally sustainable method for activating 3D-printed PLA/carbon black electrodes. Plasma exposure as short as 5–10 s significantly improved surface morphology, wettability, and electrochemical performance by selectively etching the PLA matrix and exposing conductive CB particles. Compared to conventional chemical activation methods involving DMF and NaOH, 40 s of plasma activation provided comparable results while substantially reducing processing times (vs NaOH) and eliminating the need for hazardous chemicals (such as DMF). Detailed characterization using SEM, Raman spectroscopy, XPS, contact angle analysis, and electrochemical techniques (CV and EIS) confirmed that plasma exposure enhances surface roughness, introduces oxygen-containing functional groups, improves hydrophilicity, and reduces charge transfer resistance. Electrodes plasma-activated for 40 s exhibited the most favorable electrochemical behavior in the presence of [Fe­(CN)_6_]^3–/4–^ redox indicator, with an optimal balance between surface conductivity and structural integrity. The practical applicability of the plasma-activated electrodes was demonstrated through the development, optimization, and validation of a novel and sensitive electroanalytical protocol for quantifying amlodipine in pharmaceutical formulations. This method showed excellent linearity, sensitivity, and recovery, with detection limits in the low submicromolar range and favorable repeatability across multiple measurements. In summary, these findings highlight the significant potential of atmospheric air plasma as a sustainable, rapid, scalable, and cost-effective method for enhancing the performance of 3D-printed electrochemical sensors. This work lays the foundation for future applications of such electrodes in rapid pharmaceutical screening, biomedical analyses, and point-of-care sensing.

## Experimental Section

### 3D Printing and Electrode Prototyping

FDM 3D printing was used to fabricate working electrodes for electrochemical measurements, as well as additional models for characterization techniques, including scanning electron microscopy (SEM), Raman spectroscopy, and contact angle analysis. Digital models were developed using Tinkercad software. The electrode design featured a circular head (4 mm diameter and 1 mm thickness) connected to a 46 mm long, 2 mm wide rectangular shaft, yielding a total length of 50 mm. For surface characterization, square samples (10 × 10 × 1 mm) were created for SEM and Raman spectroscopy, while rectangular samples (50 × 15 × 1 mm) were used for contact angle measurements. All designs were exported as STL files, sliced using PrusaSlicer software, and converted into G-code, which was then executed via Pronterface to control the printer. An Original Prusa i3MK3S+ FDM printer equipped with a 0.4 mm brass nozzle was used to fabricate all models. Printing was carried out using Protopasta’s Composite Conductive PLA filament, containing carbon black particles, at a nozzle temperature of 210 °C and a heated bed temperature of 60 °C. Printing parameters, including flow rate and speed, were reduced to 90% to enhance dimensional precision and ensure consistent layer bonding. During preliminary tests, these parameter modifications proved necessary to ensure a uniform surface morphology, which is directly connected to the effectiveness of surface plasma activation. These settings were selected based on manufacturer guidelines and preliminary tests. Larger models, such as those used for contact angle analysis, were printed directly onto a bed coated with a glue stick to prevent detachment and edge warping during cooling. Upon completion, the models were left to cool before removal and further processing.

### Surface Activation of 3D-Printed Electrodes

Due to the nonconductive nature of PLA matrix, surface treatment was necessary to expose conductive particles and improve electrochemical activity. Two activation methods were applied: plasma and chemical.

Atmospheric-pressure low-temperature plasma activation was conducted using a diffuse coplanar surface barrier discharge (DCSBD) system. The DCSBD incorporates comb-shaped electrodes (0.5 mm strip width, 0.5 mm gap) embedded in a 96% alumina ceramic. The discharge area is 50 × 25 mm, with the system powered by an AC source operating at 10–18 kHz and delivering up to 20 kV peak-to-peak.^68^ The total power input is 40 W, of which approximately 35 W is effectively transferred to the plasma discharge. Under these conditions, the system achieves areal and volumetric power densities of approximately 3.2 W cm^–^^2^ and 160 W cm^–^^3^, respectively, while maintaining the ceramic surface temperature below 60–70 °C.

To ensure selective treatment of the electrode’s active surface (the “head”), two layers of Kapton tape were applied to the shaft (“body”) of each electrode. This served a dual purpose: (i) shielding the shaft from unintended plasma modification, and (ii) precisely defining the distance between the electrode surface and the plasma discharge using the known thickness of the Kapton layers (∼200 μm). Only the head of the electrode was exposed to the plasma field, with both its top and bottom surfaces treated. Electrodes were manually traversed across the DCSBD plasma within the active electric field, with ambient air serving as the working gas, ensuring direct exposure^69^ to the plasma discharge throughout the treatment process. To provide a homogeneous discharge in ambient air, the system was operated at its maximum power of 35 W (approximately 3.5 W·cm^–2^). Samples were exposed for 5, 10, 20, 40, 80, and 160 s per side, corresponding to approximately 17.5–1080 J·cm^–2^ surface energy doses.

For a chemical activation, electrodes were immersed in *N,N*-dimethylformamide (DMF; 20 and 60 s) and 1 M NaOH solution (3600 and 7200 s). All immersions were conducted with the electrodes in a vertical orientation, exposing only the head to the chemical. Following each treatment, samples were rinsed with ethanol and then with distilled water, and were air-dried under ambient conditions. Care was taken to avoid physical contact with the treated surfaces to prevent unintended mechanical alteration.

Here, it is important to note the differences in activation processes. In the case of chemical activation, the entire electrode heads were immersed in solvents, and thus, the sides of the electrodes were also activated. This does not apply to plasma activation, in which only the front and back of the electrode heads were activated, but not their sides. Chemically activated electrodes should thus provide a larger surface area for electrochemical measurements.

### Characterization Methods

The surface structure and effect of activation of 3D-printed PLA/CB electrodes were studied by scanning electron microscopy (SEM, JSM-IT500HR InTouch Scope, JEOL, Japan). Raman spectra were measured using a DXR 3xi Raman Imaging Microscope (Thermo Scientific, USA) with a 532 nm excitation laser at a low power of 0.8 mW. The surface chemistry was analyzed using X-ray photoelectron spectroscopy (XPS). Measurements were carried out with an AXIS Supra spectrometer (Kratos Analytical Ltd., UK) equipped with a monochromatic Al Kα X-ray source (photon energy of 1486.6 eV) operating at 15 mA emission current and 15 kV voltage. To compensate for sample charging, an automatic electron flood gun system was employed. The spectra were recorded at a pass energy of 20 eV. Data processing was performed using CasaXPS software. A Shirley background correction was applied to account for inelastically scattered electrons. All spectra were referenced using the C–C signal at 285.0 eV. Contact angle measurements were performed by See System E (Advex Instruments, Czech Republic), and surface-free energy and its polar and dispersive components values were calculated using the Owens–Wendt model in See System Software. Two liquids with known polar and dispersive component values were used for the method: water and glycerol. Electrochemical measurements of cyclic voltammetry (CV) were performed in a three-electrode system connected to a potentiostat PalmSens4 (PalmSens, The Netherlands) controlled by the software PSTrace (PalmSens, The Netherlands). A 3D-printed PLA/CB electrode was used as the working electrode. A silver chloride electrode (Ag/AgCl/3 M KCl, BVT, Czech Republic) was used as the reference electrode, and the counter electrode was made of a platinum wire (BVT, Czech Republic). A photo of the electrochemical measurement setup, along with the connection scheme, is shown in Figure S5. CV measurements were conducted in inner-sphere redox mediator 1 mM aqueous solutions of potassium hexacyanoferrate K_3_[Fe­(CN)_6_)] in 0.1 M KCl at a scan rate of 0.1 V·s^–1^. The background measurement was performed in a supporting electrolyte containing 0.1 M KCl at a scan rate of 0.1 V·s^–1^. The size of the electroactive area was calculated based on the Randles–Ševčík equation. The values of the anodic signals measured at eight different scanning rates of 0.01, 0.025, 0.05, 0.1, 0.2, 0.3, 0.4, and 0.5 V·s^–1^ were used for the calculation. Electrochemical impedance spectroscopy (EIS) was conducted in 10 mM [Fe­(CN)_6_]^3–/4–^ containing 0.1 M KCl. A PalmSens4 potentiostat/galvanostat (PalmSens, The Netherlands) recorded Nyquist plots at +0.2 V vs Ag/AgCl from 100 kHz down to 0.1 Hz.

### Development of a Novel Electroanalytical Protocol for Drug Analysis

Voltammetric measurements were performed on the electrochemical analyzer AUTOLAB PGSTAT-101 from Metrohm Autolab B.V. (Utrecht, The Netherlands). The analyses employed a three-electrode system composed of a 3D-printed electrode (exposed to atmospheric air plasma for 40 s before use), a silver/silver chloride (Ag/AgCl) reference electrode, and a platinum counter electrode. The electrochemical analyzer was operated with NOVA software (ver. 1.11), which records the measured data and their graphical representation through various functions. Voltammograms evaluation was performed using OriginPro 8 (Origin Lab Corporation, Northampton, USA). For the preparation of BR buffers, the pHenomenal pH 1100L pH meter (VWR, Slovakia) was used to determine the exact pH value of the prepared buffers. BR buffer solution was used as the supporting electrolyte and was prepared by mixing 0.04 M H_3_BO_3_, H_3_PO_4_, and CH_3_COOH acids of a specific volume. The pH of the individual solutions in the range of 2–12 was adjusted with 0.2 M NaOH to the desired value. The prepared buffers were stored at room temperature. A stock solution of target analyte—amlodipine (AML) with a concentration of 10 mM was prepared by weighing the particular amount of amlodipine besylate (p.a. with purity of 99%, Sigma-Aldrich, Slovakia). The weighed sample was dissolved in 25 mL of methanol due to the favorable solubility profile of AML in this solvent, quantitatively transferred to a 50 mL volumetric flask, and diluted to the mark with deionized water. The prepared solution was kept in the fridge.

## Supplementary Material



## Notes

CV in 0.1 M KCl containing 1 mM [Fe­(CN)_6_]^3–/4–^ at scan rates from 0.005 to 0.5 V·s^–1^ for the plasma-activated electrodes and chemically activated electrodes; CV records showing the influence of pH in BR buffers on the current response of 1 mM AML on 40 s plasma-activated 3D-printed electrode; proposed mechanism of electrochemical oxidation of AML on 40 s plasma-activated 3D-printed electrode; DP voltammograms of 0.1 mM AML in BR buffer of pH 6 on the 40 s plasma-activated 3D-printed electrode, recorded at a constant modulation time of 50 ms and modulation amplitudes ranging from 25 to 150 mV; SW voltammograms of 0.1 mM AML in BR buffer at pH 6 on the 40 s plasma-activated 3D-printed electrode, recorded at a constant frequency of 25 Hz and amplitudes ranging from 10 to 200 mV; comparison of basic characteristics of proposed method with selected voltammetric methods for determination of AML (2021–2025); a photo of the electrochemical measurement setup and the connection scheme (PDF)
