# Correction to “Rapid Activation of 3D-Printed Carbon Electrodes by Atmospheric Air Plasma: Toward Electrochemical Drug Analysis”

**DOI:** 10.1021/acsomega.5c09132

**Published:** 2025-11-19

**Authors:** Miroslav Kováč, Katarína Gregová, Ĺubomír Švorc, František Zažímal, Tomáš Homola, Pavol Gemeiner

**Affiliations:** † Department of Graphic Arts Technology and Applied Photochemistry, Faculty of Chemical and Food Technology, 201272 Slovak University of Technology in Bratislava, Radlinského 9, 812 37 Bratislava, Slovakia; ‡ Institute of Analytical Chemistry, Faculty of Chemical and Food Technology, 201272 Slovak University of Technology in Bratislava, Radlinského 9, 812 37 Bratislava, Slovakia; § Department of Plasma Physics and Technology, Faculty of Science, 37748 Masaryk University, Kotlářská 267/2, 602 00 Brno, Czech Republic

In the published article, we have found errors in Table 3. Specifically, in three cases, these are misattributed references, and in one case, there is an error in the given data. All these errors are due to an oversight; all other data, as well as the discussion, are correct.

**Table 3:** Comparison of Different Activation Processes of 3D-Printed PLA/CB Sensors Made of Protopasta Conducting Filament on Their Duration and Electrochemical Values of Separation Potential (Δ*E*_p_) and Charge Transfer Resistance (*R*_ct_) Measured in [Fe­(CN)_6_]^3–/4–^ Redox Mediator

**Filament**	**Redox probe**	**Activation**	**Duration**	**Δ** ***E*** **(V)**	***R***_**ct**_	**Ref**
PLA/CB Protopasta	1 mM [Fe(CN)6]^3–/4–^	DCSBD Plasma	40 s	0.37	110 Ω·cm^2^	This work
	in 0.1 M KCl	DMF	20 s	0.43	125 Ω·cm^2^	
	1 mM [Fe(CN)6]^3–/4–^	Plasma O2	2 min	0.156	394 Ω·cm^2^	[46]
	in 0.1 M KCl	Plasma CO2		0.301	1915 Ω·cm^2^	
	5 mM [Fe(CN)6]^3–/4–^	Ar plasma jet	5 min	0.66	6800 Ω	[62]
	in 0.1 M KCl					
	1 mM [Fe(CN)6]^3–/4–^	Microwave	15 min	0.42	1100 Ω·cm^2^	[64]
	in 0.01 M PB	70 °C, 100W				
	1 mM [Fe(CN)6]^3–/4–^	Laser 280 mW	200 s	0.136	1410 Ω	[35]
	in 0.1 M KCl	Electrochem.				
		0.5 M NaOH				
	1 mM [Fe(CN)6]^3–/4–^	Mechanical sandpaper	∼34 min	0.092	170 Ω·cm^2^	[66]
	in 0.5 M H_2_SO_4_	30 min NaOH, ultrasonic bath				
		Electrochem.				
		0.5 M NaOH				
	1 mM [Fe(CN)6]^3–/4–^	Mechanical	400 s	0.297		[65]
	in 0.1 M KCl	Electrochem.				
		0.5 M NaOH				
	1 mM [Fe(CN)6]^3–/4–^	Mechanical	x	0.19	120 Ω	[67]
	in 0.1 M KCl	CO_2_ laser				
		Electrochem.				
		0.5 M NaOH				
	2 mM [Fe(CN)6]^3–/4–^	Air plasma	2 min	0.144	104 Ω	[63]
	in 1 M KCl	jet pen				

