# Supplementary material for: Rapid Activation of 3D-Printed Carbon Electrodes by Atmospheric Air Plasma: Toward Electrochemical Drug Analysis
Source: ACS Omega. 2025 Aug 23;10(35):40435–49. doi: 10.1021/acsomega.5c05879 (PMC12427132; doi:10.1021/acsomega.5c05879)
Supplement: Supplementary file 1 [file ao5c05879_si_001.pdf]

Supporting Information

Rapid activation of 3D-printed carbon electrodes by atmospheric air plasma: Towards electrochemical drug analysis

Miroslav Kováč[a], Katarína Gregová[a], Ľubomír Švorc[b], František Zažímal[c], Tomáš Homola[c], Pavol Gemeiner[a*]

[a]*Department of Graphic Arts Technology and Applied Photochemistry, Faculty of Chemical and Food Technology, Slovak University of Technology in Bratislava, Radlinského 9, 812 37 Bratislava, Slovakia*

[b]*Institute of Analytical Chemistry, Faculty of Chemical and Food Technology, Slovak University of Technology in Bratislava, Radlinského 9, 812 37 Bratislava, Slovakia*

[c]*Department of Plasma Physics and Technology, Faculty of Science, Masaryk University, Kotlářská 267/2, 602 00 Brno, Czech Republic*

**Corresponding author: pavol.gemeiner@stuba.sk*

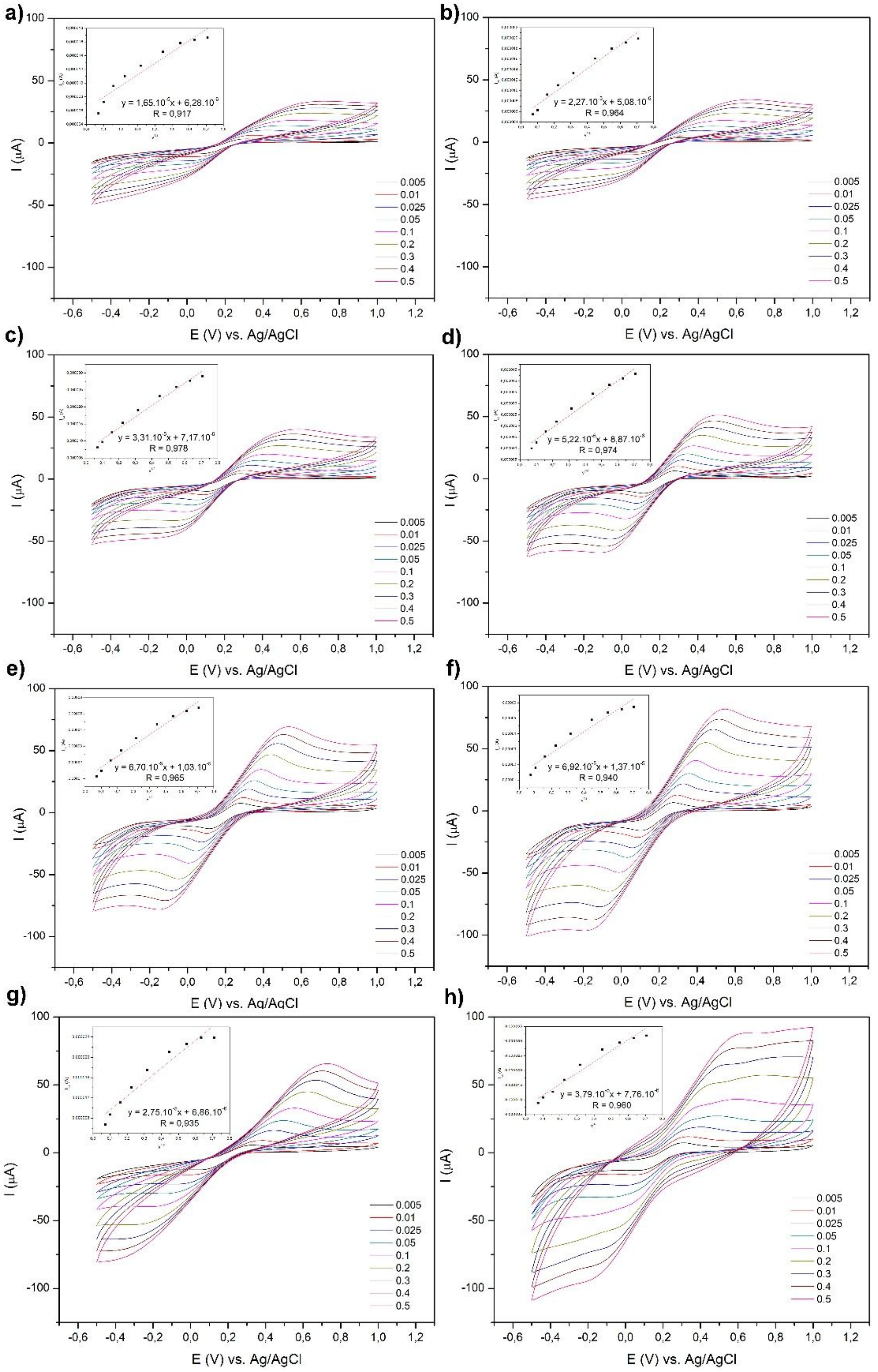

Fig. S1. Cyclic voltammograms in 0.1 M KCl containing 1 mM $[Fe(CN)_6]^{3-/4-}$at scan rates from 0.005 $V.s^{-1}$ to 0.5 $V.s^{-1}$ for the plasma-activated electrodes a) 5 s, b) 10 s, c) 20 s , d) 40 s, e) 80 s, f) 160 s and chemically activated electrodes g) NaOH 1 h, h) DMF 20 s. Inset: dependence of $I_{p.ox}$ on $v^{1/2}$.

The pH of the supporting electrolyte substantially influences the current response of the analyte and represents a fundamental factor in the optimization of experimental conditions, which is essential for achieving sensitive and consistent detection of AML. The experiment was conducted at anodic (positive) potential range, as the electrochemical activity of AML has already been investigated, and it is known that this drug undergoes an irreversible oxidation process on carbon-based electrodes. After each measurement, the surface of the working electrode was thoroughly cleaned by simple rinsing with deionized water to ensure consistency. The recorded CV records (Fig. S2) demonstrated that the electrochemical behavior of the AML was dependent on the pH of the supporting electrolyte. The results were examined based on the dependencies between the current response magnitude (I_p), the maximal peak potential (E_p), and the corresponding pH value of the buffer. The inset of Fig. S2 demonstrates that the E_p shifted to less positive values as the pH of the BR buffer increased from 2 to 8, indicating proton participation in the electrode reaction of AML on the 40s plasma-activated 3D-printed electrode. This dependence is linear over the investigated pH range and can be expressed by the following equation (Eq. 1):

$$E_p \text{ (V)} = (1.215 \pm 0.008) - (0.024 \pm 0.002) \times \text{pH} \qquad R^2 = 0.988 \qquad (1)$$

Based on the experimental observations (a slope of 0.024 V/pH compared to the Nernstian theoretical value of 0.059 V/pH), the electrochemical oxidation of AML appears to be proton-dependent, involving the loss of two electrons coupled with the removal of one proton. The proposed mechanism is shown in Scheme S1. The electrode reaction of AML probably begins with the oxidation of the 1,4-dihydropyridine (1,4-DHP) ring in the first step, involving a two-electron and one-proton transfer to form a pyridinium cation. The proton bound to the nitrogen atom of the 1,4-DHP ring is considered to be responsible for the oxidation mechanism and represents the rate-determining step. In the second step, an aromatic pyridine ring may be formed through the loss of an additional proton from the pyridinium cation. This postulate is in good agreement with the result reported on the electrochemical oxidation of AML on a boron-doped diamond electrode[1]. At higher pH values (9-12), no distinct current signals of AML were observed (these current responses were omitted from Fig. S2). Among the buffer solutions used, the highest value (40.9 mA) at a potential of +1.07 V was achieved at pH 6 (thick purple curve in Fig. S2). For this reason, the BR buffer of pH 6 was chosen as the supporting electrolyte for the subsequent measurements.

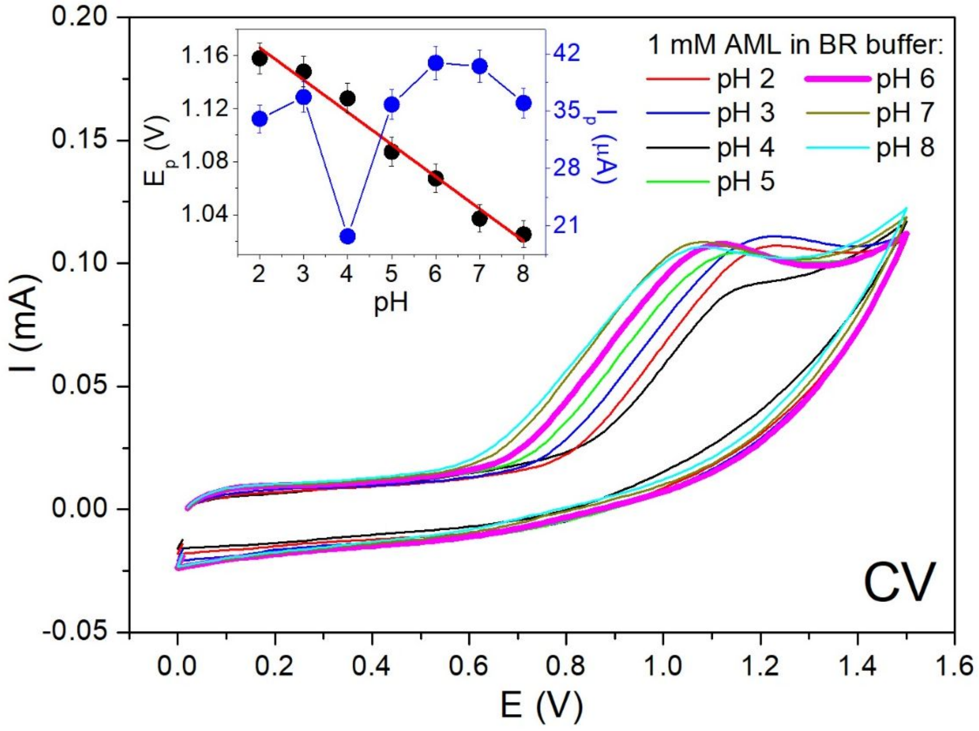

Fig. S2. CV records showing the influence of pH in BR buffers on the current response of 1 mM AML on 40s plasma-activated 3D-printed electrode, recorded at a scan rate of 100 mV/s. Inset: dependence I_p and E_p on pH.

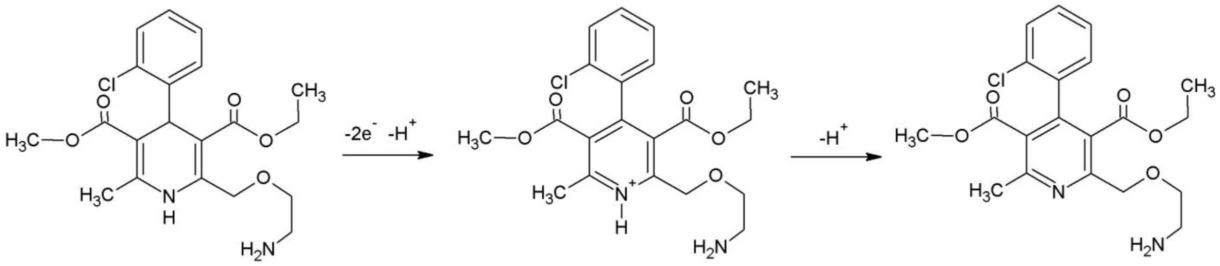

Scheme S1. Proposed mechanism of electrochemical oxidation of AML on 40s plasma-activated 3D-printed electrode.

The following section focused on the optimization of instrumental parameters for pulse voltammetric techniques, namely differential pulse voltammetry (DPV) and square-wave voltammetry (SWV). Proper adjustment of variables such as modulation time and modulation amplitude for DPV, and frequency and amplitude for SWV, was essential for enhancing the oxidation peak of 0.1 mM AML in BR buffer of pH 6 on the 3D-printed electrodes while reducing background noise. The optimal experimental conditions were chosen based on a balance between maximum current response and its stability for reliable detection of AML in further analyses. In the case of instrumental parameters of DPV, the modulation amplitude was adjusted between 10 and 150 mV in the search for a favourable value. Modulation time was

kept constant at 50 ms, as predefined by software settings. Fig. S3 demonstrates that with increasing modulation amplitude, the current response of AML also increases. However, at elevated values, signal instability and distortions were observed. The 10 mV amplitude was excluded, as the signal was indistinguishable from the noise. Based on the observed data, a modulation amplitude of 100 mV (thick green curve in Fig. S3) was chosen because of the balance between signal intensity and minimal distortion. The subsequent step was to find the optimal modulation time value by varying it from 10 to 150 ms, while the modulation amplitude remained constant at 25 mV. The corresponding voltammograms are shown in the inserted image in the top left corner (Fig. S3). The values of 10 ms and 25 ms were excluded due to indistinct and distorted peaks of AML. With a modulation time of 50 ms, a higher peak, an elevated baseline current and increased noise were detected. An increase in the value led to a decrease in oxidation peak intensity but produced a more stable signal. At 150 ms (thick purple curve in the inserted image of Fig. S3), the lowest background current and a distinct signal of AML were obtained, making it the optimal choice for further measurements.

SWV parameter optimization followed the same procedure as for DPV. The particular records are shown in Fig. S4. The initial step involved determining the optimal amplitude value within the range from 10 to 200 mV at a constant frequency of 25 Hz. As the amplitude value increased, an increase in the oxidation peaks of AML was noted. Among the tested values, an amplitude of 75 mV (thick green curve in Fig. S4) achieved the best balance, producing a well-defined peak with sufficient intensity and, therefore, was chosen as the optimal value. As the final step in the optimization, the frequency between 10 and 200 Hz was evaluated, keeping the amplitude constant at 20 mV. Since no oxidation peak of AML was recorded at 150 and 200 Hz, their measurements were omitted from the records. With rising frequency, peak currents diminish, and peak shapes become less distinct. The 10 Hz frequency (thick black curve in the inserted image of Fig. S4) produced the most defined peak with the highest current response, making this value an optimal choice for AML detection with improved sensitivity.

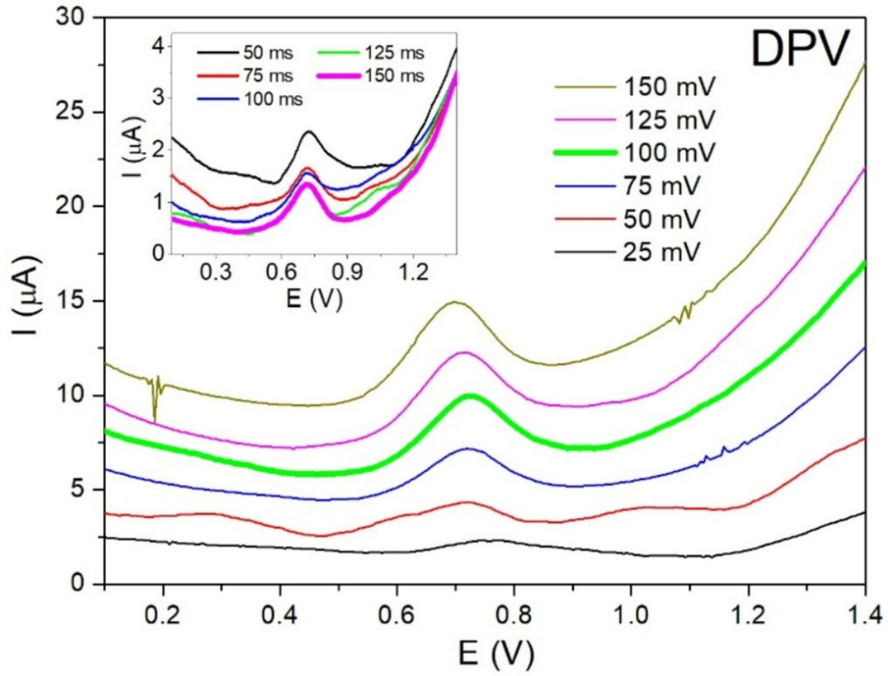

Fig. S3. DP voltammograms of 0.1 mM AML in BR buffer of pH 6 on the 40s plasma-activated 3D-printed electrode, recorded at a constant modulation time of 50 ms and modulation amplitudes ranging from 25 mV to 150 mV. Inset: DP voltammograms of 0.1 mM AML in BR buffer of pH 6 on the 3D-printed electrode, recorded at a constant modulation amplitude of 25 mV and modulation times ranging from 50 ms to 150 ms.

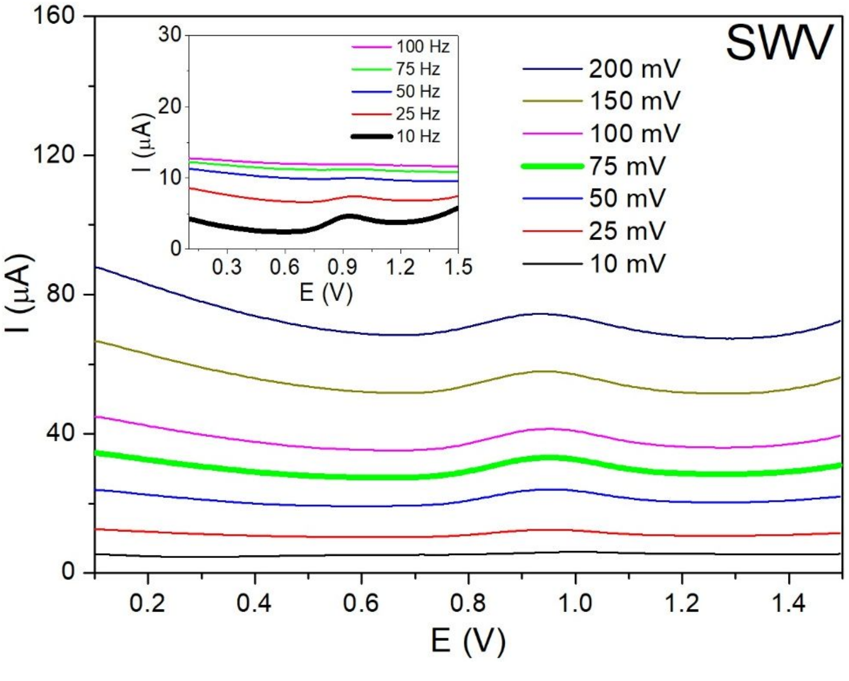

Fig. S4. SW voltammograms of 0.1 mM AML in BR buffer of pH 6 on the 40s plasma-activated 3D-printed electrode, recorded at a constant frequency of 25 Hz and amplitudes ranging from 10 mV to 200 mV. Inset: SW voltammograms of 0.1 mM AML in BR buffer of pH 6 on the 3D-printed electrode, recorded at a constant amplitude of 20 mV and frequencies ranging from 10 Hz to 100 Hz.

Tab. S1 Comparison of basic characteristics of proposed method with selected voltammetric methods for determination of AML (2021-2025).

**Electrode**	**Supporting electrolyte**	**Technique**	**E_p (V)**	**LCR (μM)**	**LOD (μM)**	**Analyzed sample**	**Ref.**
GNRs-GO-fCNTs/GCE	PB, pH 4.8	DPV	+0.7	0.01 – 1	0.003	pharmaceuticals, blood serum and plasma	[2]
CuO-NiO/IL/CPE	PB, pH 9.0	DPV	+0.5	0.1 – 100	0.06	pharmaceuticals, human urine, blood plasma	[3]
MESA/CPE	PB, pH 8.0	DPV	+0.2	1 – 20	0.4	pharmaceuticals	[4]
Au-NPs@rGO/CPE	PB, pH 6.0	DPV	+0.8	2 – 14	0.36	pharmaceuticals	[5]
HMDE	BR, pH 8.0	SWV	-	14.3 – 40.4	1.2	pharmaceuticals	[6]
Zn-Ca_2CuO_3/GCE	PB, pH 7.0	SWV	+0.8	0.8 – 354	0.11	pharmaceuticals, human urine	[7]
Plasma-activated 3D-printed electrode	BR, pH 6.0	DPV	+0.7	0.7 – 10	0.09	pharmaceuticals	This work

Abbreviations: AML – amlodipine, Au-NPs@rGO/CPE – gold nanoparticle/reduced graphene oxide modified carbon paste electrode, BR – Britton-Robinson buffer, CuO-NiO/IL/CPE – CuO-NiO nanocomposite/ionic liquid modified carbon paste electrode, DPV – differential pulse voltammetry, E_p – peak potential, GNRs-GO-fCNTs/GCE – gold nanorods-graphene oxide-functionalized carbon nanotubes nanocomposite modified glassy carbon electrode, HMDE – hanging mercury dropping electrode, LCR – linear concentration range, LOD – limit of detection, MESA/CPE – mesalazine modified carbon paste electrode, PB – phosphate buffer, SWV – square-wave voltammetry, Zn-Ca_2CuO_3/GCE – Zn-doped Ca_2CuO_3 nanoparticles modified glassy carbon electrode

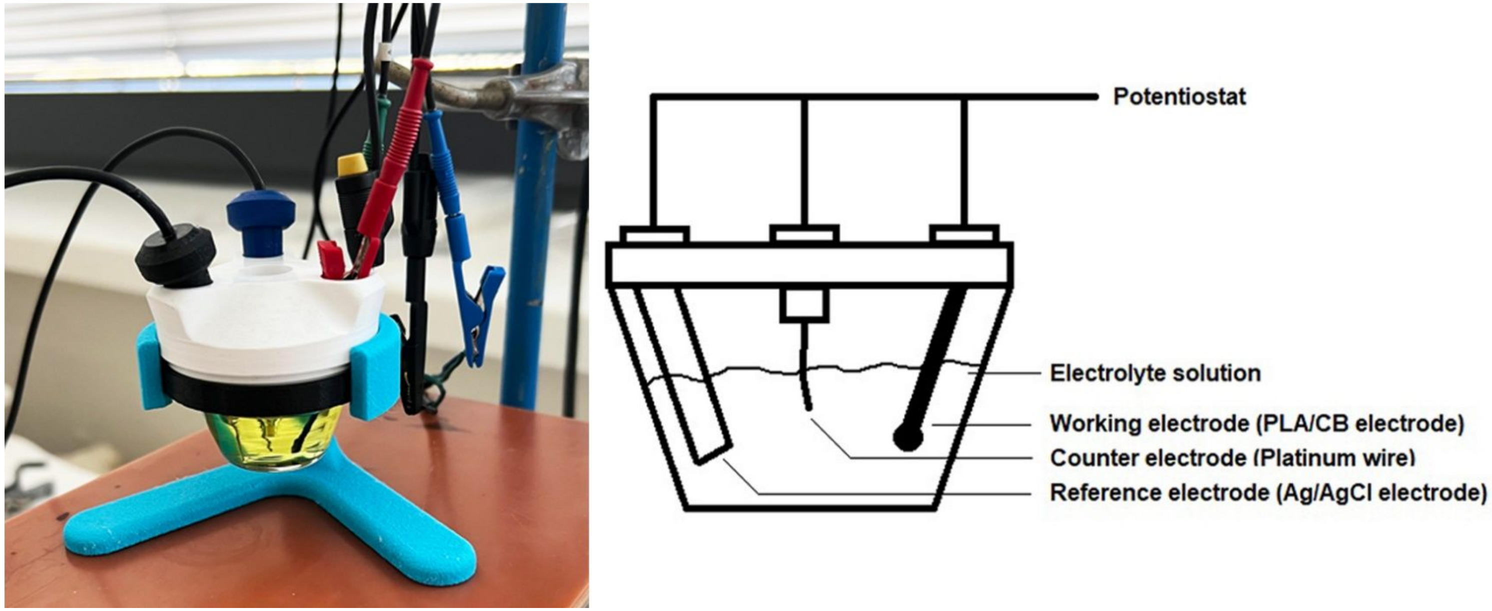

Fig. S5. A photo of the electrochemical measurement setup and the connection scheme.

References

[1]Švorc, Ľ.; Cinková, K.; Sochr, J.; Vojs, M.; Michniak, P.; Marton, M. Sensitive electrochemical determination of amlodipine in pharmaceutical tablets and human urine using boron-doped diamond electrode. *Journal of Electroanalytical Chemistry*, **2014**, *728*, 86–93.

[2]Arvand, M.; Kaykhaii, M.; Ashrafi, P.; Hemmati, S. An electrochemical interface for direct analysis of amlodipine in tablets and human blood samples. *Materials Science and Engineering: B*, **2021**, *263*, 114868.

[3]Firouzi, M.; Giahi, M.; Najafi, M.; Homami, S.S.; Mousavi, S.H.H. Electrochemical determination of amlodipine using a CuO-NiO nanocomposite/ionic liquid modified carbon paste electrode as an electrochemical sensor. *Journal of Nanoparticle Research*, **2021**, *23*, 82.

[4]Firouzi, M.; Nafaji M. Mesalazine Modified Carbon Paste Electrode for Voltammetric Determination of Amlodipine. *Journal of Applied Chemical Research*, **2023**, *17*, 82–95.

[5]Marzouk, H.M.; El-Hanboushy, S.; Mahmoud, A.M.; Fayez, Y.M; Abdelkawy, M.; Lofty, H.M. A novel electrochemical sensor based on reduced graphene oxide decorated with gold nanoparticles for voltammetric sensing of amlodipine in human urine. *Journal of Applied Electrochemistry*, **2025**, *55*, 813–824.

[6]Palabıyık, I.M.; Dogan, A.; Süslü, I. Simultaneous Determination of Amlodipine and Irbesartan in their Pharmaceutical Formulations by Square-Wave Voltammetry. *Combinatorial Chemistry & High Throughput Screening*, **2022**, *25*, 241–251.

[7]Veerapandi, G.; Sekar, C. Binder-free and efficient voltammetric sensor based on Zn-Ca_2CuO_3 nanoparticles for simultaneous determination of amlodipine, acetaminophen, and ascorbic acid in hypertension patients. *Microchimica Acta*, **2024**, *9*, 409.
